# Code-Assisted Discovery of TAL Effector Targets in Bacterial Leaf Streak of Rice Reveals Contrast with Bacterial Blight and a Novel Susceptibility Gene

**DOI:** 10.1371/journal.ppat.1003972

**Published:** 2014-02-27

**Authors:** Raul A. Cernadas, Erin L. Doyle, David O. Niño-Liu, Katherine E. Wilkins, Timothy Bancroft, Li Wang, Clarice L. Schmidt, Rico Caldo, Bing Yang, Frank F. White, Dan Nettleton, Roger P. Wise, Adam J. Bogdanove

**Affiliations:** 1 Department of Plant Pathology and Microbiology, Iowa State University, Ames, Iowa, United States of America; 2 Department of Plant Pathology and Plant-Microbe Biology, Cornell University, Ithaca, New York, United States of America; 3 Bioinformatics and Computational Biology Graduate Program, Iowa State University, Ames, Iowa, United States of America; 4 Department of Statistics, Iowa State University, Ames, Iowa, United States of America; 5 Genetics Development and Cell Biology, Iowa State University, Ames, Iowa, United States of America; 6 Department of Plant Pathology, Kansas State University, Manhattan, Kansas, United States of America; 7 Corn Insects and Crop Genetics Research, USDA-ARS, Iowa State University, Ames, Iowa, United States of America; Chinese Academy of Sciences, China

## Abstract

Bacterial leaf streak of rice, caused by *Xanthomonas oryzae* pv. oryzicola (Xoc) is an increasingly important yield constraint in this staple crop. A mesophyll colonizer, Xoc differs from *X. oryzae* pv. oryzae (Xoo), which invades xylem to cause bacterial blight of rice. Both produce multiple distinct TAL effectors, type III-delivered proteins that transactivate effector-specific host genes. A TAL effector finds its target(s) via a partially degenerate code whereby the modular effector amino acid sequence identifies nucleotide sequences to which the protein binds. Virulence contributions of some Xoo TAL effectors have been shown, and their relevant targets, susceptibility (*S*) genes, identified, but the role of TAL effectors in leaf streak is uncharacterized. We used host transcript profiling to compare leaf streak to blight and to probe functions of Xoc TAL effectors. We found that Xoc and Xoo induce almost completely different host transcriptional changes. Roughly one in three genes upregulated by the pathogens is preceded by a candidate TAL effector binding element. Experimental analysis of the 44 such genes predicted to be Xoc TAL effector targets verified nearly half, and identified most others as false predictions. None of the Xoc targets is a known bacterial blight *S* gene. Mutational analysis revealed that Tal2g, which activates two genes, contributes to lesion expansion and bacterial exudation. Use of designer TAL effectors discriminated a sulfate transporter gene as the *S* gene. Across all targets, basal expression tended to be higher than genome-average, and induction moderate. Finally, machine learning applied to real vs. falsely predicted targets yielded a classifier that recalled 92% of the real targets with 88% precision, providing a tool for better target prediction in the future. Our study expands the number of known TAL effector targets, identifies a new class of *S* gene, and improves our ability to predict functional targeting.

## Author Summary

Many crop and ornamental plants suffer losses due to bacterial pathogens in the genus *Xanthomonas*. Pathogen manipulation of host gene expression by injected proteins called TAL effectors is important in many of these diseases. A TAL effector finds its gene target(s) by virtue of structural repeats in the protein that differ one from another at two amino acids that together identify one DNA base. The number of repeats and those amino acids thereby code for the DNA sequence the protein binds. This code allows target prediction and engineering TAL effectors for custom gene activation. By combining genome-wide analysis of gene expression with TAL effector binding site prediction and verification using designer TAL effectors, we identified 19 targets of TAL effectors in bacterial leaf streak of rice, a disease of growing importance worldwide caused by *X. oryzae* pv. oryzicola. Among these was a sulfate transport gene that plays a major role. Comparison of true vs. false predictions using machine learning yielded a classifier that will streamline TAL effector target identification in the future. Probing the diversity and functions of such plant genes is critical to expand our knowledge of disease and defense mechanisms, and open new avenues for effective disease control.

## Introduction

Bacterial leaf streak of rice (*Oryza sativa*), caused by *Xanthomonas oryzae* pv. oryzicola (Xoc), and bacterial blight of rice, caused by the closely related *Xanthomonas oryzae* pv. oryzae (Xoo) are important constraints to production of this staple crop in many parts of the world. Yield losses as high as 50% for blight and 30% for leaf streak have been documented [1]. Leaf steak in particular appears to be growing in importance, as high-yielding but susceptible hybrid varieties of rice are increasingly adopted (C. Vera-Cruz and G. Laha, personal communications). Xoc enters through leaf stomata or wounds and interacts with mesophyll parenchyma cells to colonize the mesophyll apoplast, causing interveinal, watersoaked lesions that develop into necrotic streaks. Quantitative trait loci for resistance to leaf streak have been characterized [2], but native major gene resistance has yet to be identified. In contrast, Xoo typically enters through hydathodes or wounds and travels through the xylem, interacting with xylem parenchyma cells through the pit membranes, and typically resulting in wide necrotic lesions along the leaf margins or following veins down the center of the leaf. Only in later stages of disease development does Xoo colonize the mesophyll. Also in contrast to leaf streak, roughly 30 independent genes for resistance (R) to blight have been identified and seven molecularly characterized [3], [4]. The basis for the distinct tissue specificities of Xoc and Xoo and the disparity in known host resistance, despite the genetic similarity of the two pathogens, is not known.

Virulence of Xoo, and of *Xanthomonas* that infect citrus, cotton, or pepper, is influenced by transcription activator-like (TAL) effectors [5]–[15]. Widespread in *Xanthomonas*, TAL effectors are proteins delivered into the plant cell via type III secretion (T3S) that transactivate effector-specific host genes [16], [17]. If activation is important for disease, the target is considered a susceptibility (*S*) gene [9]. Individual Xoo strains harbor multiple, distinct TAL effector (*tal*) genes [8], and several bacterial blight *S* genes have been identified. The first of these were *Os8N3* (a sugar transporter gene family member also and hereafter referred to as *OsSWEET11*), the bZIP transcription factor *OsTFXI*, and the transcription initiation factor *TFIIAγI*, upregulated respectively by TAL effectors PthXo1, PthXo6, and PthXo7 of Xoo strain PXO99^A^ [9], [10]. More recently, the closely related *OsSWEET11* paralog *OsSWEET14* (also *Os11N3*) was discovered to be an *S* gene targeted by several distinct TAL effectors from other strains [11], [18], [19]. A third close paralog upregulated during infection by some strains, *OsSWEET12*, also functions as an *S* gene, though a TAL effector that upregulates it has not yet been reported [19], [20]. The recessive blight *R* genes *xa13* and *xa25* are promoter variant alleles of *OsSWEET11* and *OsSWEET12*, respectively, that are not activated by the corresponding TAL effector (or presumed TAL effector in the case of *OsSWEET12*) [9], [20]. Some TAL effectors induce host resistance by transcriptionally activating a type of dominant *R* gene that triggers local cell death when expressed, for example the archetypal TAL effector AvrBs3 from the pepper pathogen *X. euvesicatoria* [21], which activates the pepper *Bs3* gene for resistance to bacterial spot [17], and the Xoo effector AvrXa27, from strain PXO99^A^, which induces the rice *R* gene *Xa27* [22]. Like Xoo, Xoc strains harbor multiple *tal* genes [8], [23]. However, though the T3S system through which TAL effectors travel is required for leaf streak [24], the role of Xoc TAL effectors in the disease is uncharacterized, and no leaf streak *S* genes have been identified.

TAL effectors find their targets via a structurally modular mechanism that allows prediction of DNA specificity and customization to target nucleotide sequences of choice [25]–[29]. The modules are tandem repeats of a 33–35 amino acid sequence, exhibiting polymorphism at residues 12 and 13, together called the repeat variable diresidue (RVD). Different RVDs were shown computationally and experimentally, and later structurally to each specify a single nucleotide through direct interaction with (or exclusion of other bases by) the residue 13 side chain, such that the string of RVDs presented by the repeats “encodes” the sequence of the so-called TAL effector binding element (EBE) on the DNA [25], [26], [30], [31]. The RVD nucleotide associations observed in nature are not strictly one to one, however [26]. Indeed, all known natural EBEs contain one or more mismatches to the corresponding TAL effector RVD sequence, a mismatch being a base different from the one most commonly associated with the RVD. Furthermore, some RVDs have dual or even entirely lax specificity. So, the TAL effector-DNA binding code is partially degenerate, rendering target prediction probabilistic [26], [32]. Finally, EBEs in nature are almost all directly preceded by a 5′ thymine (T) that has been shown, in the few studied cases, to be important for TAL effector-driven gene activation as well as full affinity DNA binding [33]–[35]. The single known exception, EBE_TalC_ in the promoter of *OsSWEET14*, displays a cytosine (C). Although the effect of substituting a T was not tested directly, a perfect match EBE for TalC, with a T at base 0 and corrected mismatches at two other locations, indeed showed higher activity [13]

In this study, we sought to better understand bacterial leaf streak in relation to bacterial blight, particularly with an eye toward identifying determinants of tissue specificity, and to examine the roles of Xoc TAL effectors in disease. We began by comparing transcription profiles in Xoc-, Xoo-, and mock-inoculated plants by microarray analysis. We then combined the transcriptomic data with computational identification of candidate EBEs to predict TAL effector targets, and carried out experiments to differentiate real from falsely predicted ones. Screening a TAL effector mutant library of Xoc, we next identified a TAL effector that plays a major role in virulence, and we discriminated from among its two targets the first known *S* gene for leaf streak, in part by using designer TAL effectors to independently activate the genes. Using our complete list of newly discovered targets as well as the previously identified Xoo targets represented in our dataset, we next examined general characteristics of TAL effector driven gene expression. Finally, in an attempt to better discriminate real targets from falsely predicted ones in the future, prior to experimentation, we used machine learning to train a classifier on primary and contextual features of EBEs in the respective groups. Our results provide new insight into bacterial leaf streak, increase the number of known natural TAL effector combinations by 20, identify a new class of *S* gene, and advance our understanding of and ability to predict functional targeting by TAL effectors.

## Results

### *X. oryzae* pv. oryzicola BLS256 and *X. oryzae* pv. oryzae PXO99^A^ induce largely different gene expression changes in rice leaves

We initially set out to determine whether there are differences in host genome-wide expression patterns during bacterial leaf streak vs. bacterial blight that might help to explain the different tissue specificity of Xoc and Xoo. Using a vacuum infiltration approach developed from a dipping method we showed previously to be effective for both pathovars [36], we inoculated rice (cv. Nipponbare) plants *en masse* with Xoc strain BLS256 (hereafter Xoc refers to this strain unless otherwise specified), Xoo strain PXO99^A^ (likewise), or a mock inoculum, harvested leaves at 2, 4, 8, 24, and 96 hours thereafter, and quantified transcript levels in these leaves for the roughly 56,000 annotated rice genes in parallel using the Affymetrix GeneChip Rice Genome Array [37]. We focused our analysis on patterns of expression across the time course rather than expression levels at a particular time point and examined three pairwise comparisons, Xoc vs. mock, Xoo vs. mock, and Xoc vs. Xoo (see Materials and Methods).

**Figure 1:**
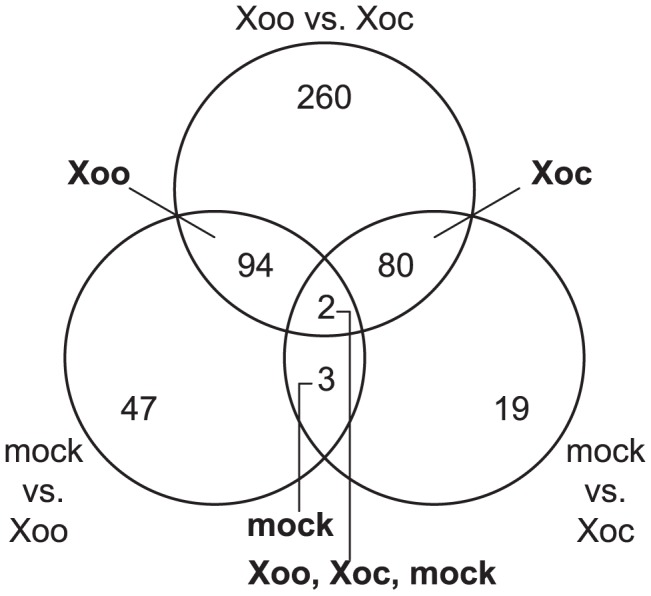
Rice transcriptional responses to *Xanthomonas oryzae* pv. oryzicola BLS256 (Xoc) or *X. oryzae* pv. oryzae PXO99^A^ (Xoo). Distribution of genes differentially expressed over a 96(see Materials and Methods) in response to either strain relative to a mock inoculation is shown. Each circle of the Venn diagram represents a different pairwise comparison of treatments, as indicated in non-bold text. Results are based on mixed linear model analysis using four biological replicates for each time point of the study and an estimated false discovery rate of 0.3. The intersections represent the genes differentially expressed uniquely in response to the different treatments, indicated in bold text. Note that differentially expressed uniquely in response to mock means differentially expressed similarly in Xoc and Xoo relative to mock, and differentially expressed uniquely in response to all three treatments means differentially expressed both in Xoc and Xoo relative to mock, but also differentially between Xoc and Xoo. Also, since differential expression in a given pairwise comparison is determined using a statistical cutoff, transitive predictions, i.e., A = B and B = C, therefore A = C, may not hold.

**Figure 2:**
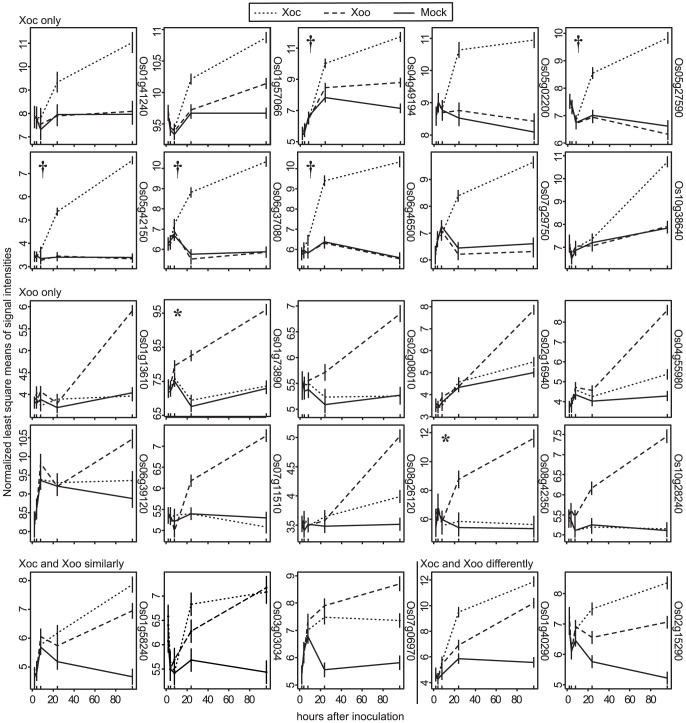
Expression patterns of the most significantly differentially expressed rice genes. Normalized least square means of signal intensities (y-axis) at 2, 4, 8, 24, and 96 h after inoculation (x-axis) with *X. oryzae* pv. oryzicola BLS256 (Xoc), *X. oryzae* pv. oryzae strain PXO99^A^ (Xoo) or mock control are plotted for the genes most significantly differentially expressed relative to mock uniquely in response to Xoc (Xoc only), uniquely in response to Xoo (Xoo only), similarly in response to Xoc and Xoo (Xoc and Xoo similarly), and differently in response to Xoc and Xoo (Xoc and Xoo differently). Where two probe sets correspond to the same gene, the one with the lower *q*-value was selected for display. Locus IDs are given at right, omitting the prefix “LOC_Os”. Results were derived from a mixed linear model analysis with four replicates. Vertical bars represent standard error. Asterisks mark previously identified targets of Xoo TAL effectors, *TFIIaγ1*(*Os01g73890*) and *OsSWEET11* (*Os08g42350*), activated by PthXo7 and PthXo1, respectively. Daggers flag Xoc TAL effector targets discovered in this study.

A total of 505 genes showed significantly different expression profile patterns (*q*≤0.3; see Materials and Methods) in one or more of the pairwise comparisons (Figure 1). Eighty and 94 genes were differentially expressed uniquely in response to Xoc or Xoo, respectively (Figure 1; Table S1 and Table S2). Only five genes were differentially expressed both in response to Xoc and Xoo relative to mock: three similarly between Xoc- and Xoo- and two with different patterns in Xoc- vs. Xoo-inoculated plants (Figure 1; Table S3). Strikingly, all of the statistically significantly differentially expressed genes showed patterns of upregulation in response to Xoc or Xoo. Expression patterns of the ten or fewer most significantly differentially expressed genes in response to Xoc, Xoo, or both are shown in Figure 2.

Singular enrichment analysis [38] of gene ontology (GO) for all Xoc- and Xoo-upregulated genes revealed broad differences in the major categories represented (Table S4 and Table S5). Six significant GO terms were identified for Xoc-induced genes. Four of these are categorized under biological processes and include coenzyme metabolic, cofactor metabolic, sulfur metabolic and, cellular amino acid derivative metabolic processes. The other two, catalytic and oxidoreductase activities, are grouped under molecular function (Table S4). For Xoo-induced genes, the significant terms all fall within the cellular component category, including membrane-bounded vesicle, vesicle, cytoplasmic membrane-bounded vesicle, and cytoplasmic vesicle (Table S5). The most abundant ontology category for genes induced by Xoc was catalytic activity, and included several glutathione S-transferase and oxidase genes (Table S4). These were part of a large group of Xoc-induced genes, distributed among several categories, with annotations that suggest roles in reactive oxygen species detoxification and redox status control (assembled together in Table S6).

Among the complete list of Xoo-induced genes are each of the bacterial blight *S* genes previously reported to be induced by PXO99^A^ TAL effectors, *OsSWEET11* (*Os08g42350*), *OsTFXI* (*Os09g29820*), and *TFIIAγI* (*Os01g73890*) (Table S2 and Table S7). Notably, none of these three genes nor any of the Os*SWEET11* paralogs reported to function as bacterial blight *S* genes [11], [19], [20] was activated following inoculation with Xoc.

Thus, host genome wide expression patterns during bacterial leaf streak vs. bacterial blight are almost completely different.

### The most significant gene expression changes depend on bacterial type III secretion

**Figure 3:**
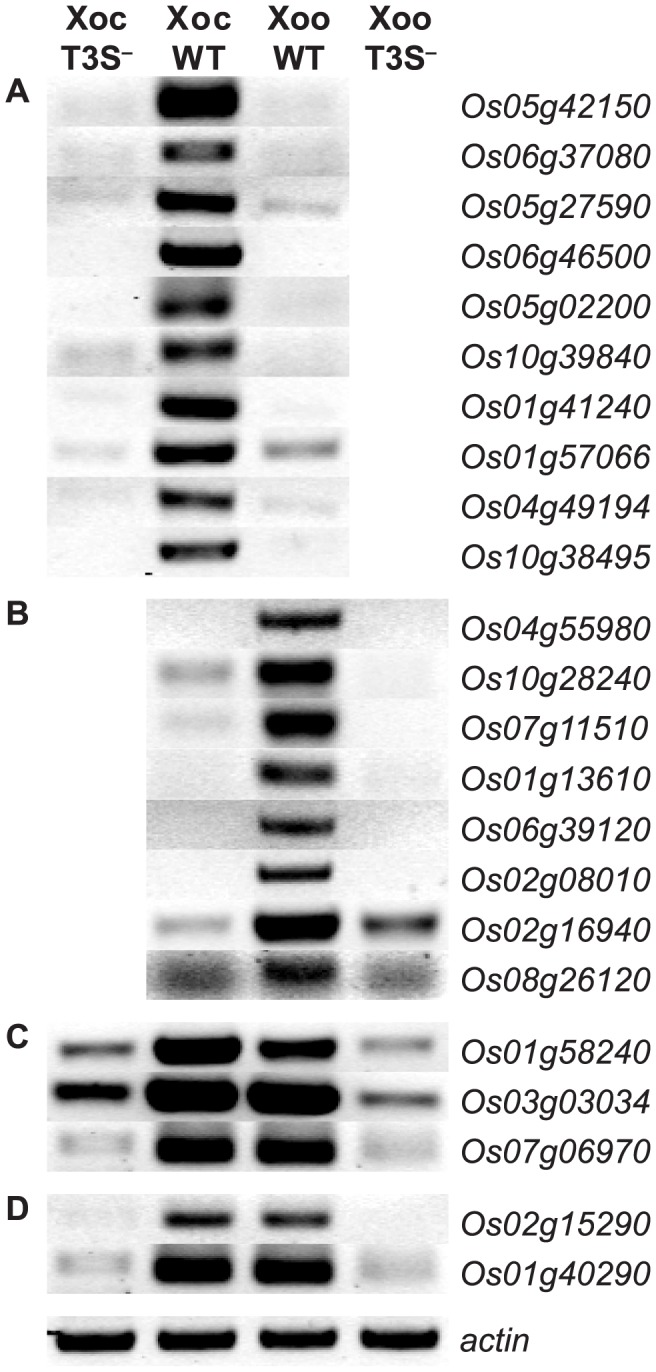
Type III secretion system dependence of the most significant rice gene expression changes. RT-PCR results reflecting transcript abundance are shown for rice genes identified by GeneChip expression analysis as the ten (or fewer) most significantly differentially expressed in response to (A) *X. oryzae* pv. oryzicola BLS256 (Xoc), (B) *X. oryzae* pv. oryzae strain PXO99^A^ (Xoo), (C) Xoc and Xoo similarly, or (D) Xoc and Xoo to different extents. Leaf samples were harvested at 36 hours after inoculation with wild-type strains or with the type III secretion (T3S^−^) deficient derivatives. RT-PCR results for previously reported Xoo-induced genes, *OsSWEET11* and *TFIIAγ1* [9], [10], are omitted. An actin gene (*Os04g57210*) that is not differentially expressed was used as a reference for relative transcript abundance across samples. The experiment was repeated twice and yielded the same results.

The TAL effector inventories in Xoc and Xoo are entirely distinct. Xoc harbors 26 unique, intact TAL effector genes and Xoo 14, with no shared predicted EBEs based on RVD sequences [23], [39]. The inventories of predicted non-TAL type III effectors in Xoc and Xoo are similar, but six effector genes present in Xoc are absent from or pseudogenized in Xoo and several minor polymorphisms exist among the shared genes [23]. As a first step to determine the extent to which differences in TAL or other type III effector content might account for the differences in rice global transcription patterns we observed, we asked whether T3S is required for induction of the top ten rice genes most significantly induced uniquely following inoculation with Xoc, the top ten induced by Xoo, and all five induced in common by both strains. We compared, by RT-PCR, transcript accumulation after inoculation with the wild-type strains or with T3S-deficient derivatives BLS256*hrcC^−^* [24] and PXO99^A^ME7 [9]. Induction of each gene required bacterial T3S (Figure 3 and [9], [10]).

Among the top ten Xoo-induced genes are the TAL effector targets *OsSWEET11* (*Os08g42350*) and *TFIIaγ1* (*Os01g73890*). The patterns of induction of each of the top Xoc- or Xoo-induced genes revealed by the genome-wide expression analysis described in the previous section vary, but some are similar to that of *OsSWEET11* and *TFIIaγ1* (Figure 2). This similarity and the T3S-dependence of expression suggested that some of these and perhaps others in the complete lists of induced genes are targets of TAL effectors.

### Many upregulated genes are predicted targets of TAL effectors

To identify TAL effector targets, we first used the scoring function we developed previously based on observed RVD-nucleotide association frequencies [26], [32] to scan *in silico* all annotated rice gene promoters (the promoterome) [32] for candidate EBEs for any of the 40 total TAL effectors present in Xoc and Xoo [23], [39]. Some of these TAL effectors have new RVDs whose specificities have not been characterized. The scoring function by default treats new RVDs as wild cards, equally likely to specify any base. However, since structural studies revealed that the second residue of each RVD makes the base-specific contacts while the first stabilizes the inter-helical loop that projects that second residue into the major groove of the DNA [30], [31], we used the specificities of common RVDs for any new RVDs that share the same second position residue. These were limited to two RVDs found in Xoc TAL effector Tal2g, ‘SN’ for which we substituted nucleotide association frequencies of ‘NN’, and ‘YG’ for which we substituted those of ‘NG’. Candidate EBEs were required to be directly preceded by a T at the 5′ end and, for each TAL effector, to score below a cutoff calculated based on the distribution of scores for that effector (see Materials and Methods). This list was then cross-referenced to the GeneChip expression data, and genes with one or more candidate EBEs in the promoter that were also induced following inoculation with the corresponding strain were retained as predicted targets (Table S7).

Thirty-five of these are genes induced by Xoc (three of the 35 are also induced by Xoo), and they collectively contain candidate EBEs for 19 out of the 26 Xoc TAL effectors. Twenty-nine are genes induced by Xoo (five are also induced by Xoc), and they together contain putative EBEs for all 14 of the unique Xoo TAL effectors (Tal7a and 7b are identical to Tal8a and 8b, respectively). The latter include each of the three previously demonstrated targets of Xoo (i.e., PXO99^A^) TAL effectors in Nipponbare, *OsSWEET11* targeted by PthXo1, *OsTFXI* targeted by PthXo6, and *TFIIAγI* targeted by PthXo7 [9], [10], [40] (the AvrXa27-activated allele of *Xa27* is not present in Nipponbare). Among the five genes induced in common by Xoc and by Xoo, two were predicted to be targeted by a TAL effector from Xoo but not by one from Xoc (*Os01g58240* by Tal4 and *Os01g40290* by Tal7b/8b of Xoo). In the other three, sequence distinct, candidate EBEs for one or more TAL effectors from each strain were found in the promoters (EBEs for Tal2c and Tal3b of Xoc and AvrXa27 and Tal9b of Xoo in *Os03g03034*, for Tal1c and Tal3a of Xoc and Tal9a of Xoo in *Os07g06970*, and for Tal5a and Tal11a of Xoc and Tal9e of Xoo in *Os02g15290*).

Of the 35 total genes induced by Xoc that harbor a candidate EBE for an Xoc TAL effector, eight harbor EBEs for more than one. Likewise, of the 29 Xoo-induced genes that match an Xoo TAL effector, four genes contain EBEs for multiple Xoo TAL effectors. These results suggest for both pathovars a partial redundancy among effectors for some targets. The Xoc-induced gene *Os06g14750* and the Xoo-induced gene *Os07g11510* contain overlapping candidate EBEs for three TAL effectors each from those strains, Tal2a, Tal1c, and Tal11b, and PthXo6, Tal2a, and Tal5a, respectively.

The number of predicted targets for individual TAL effectors varies. In the case of Xoc, we identified five predicted targets each for Tal3b and Tal6, and one of the predicted Tal6 targets, *Os12g42970*, harbors two candidate Tal6 EBEs. Five Xoc TAL effectors, Tal2c, Tal5a, Tal8, Tal9b and Tal11b, have only one predicted target each. For Xoo, we predicted seven targets for PthXo6 and one target each for PthXo1, PthXo7, Tal6a, Tal7a/8a, Tal9d, and Tal9e. AvrXa27 had five predicted targets, two of which, Os06g03080 and *Os06g03120*, are paralogs nearly identical in their coding sequences and both represented by a single probeset. The promoters of these genes share the same AvrXa27 EBE (one of two AvrXa27 EBEs in *Os06g03120*), but are otherwise distinct.

In sum, all but a few of the TAL effectors of Xoc and Xoo have candidate binding sites in a gene upregulated by that strain; a total of 61 out of 179, or roughly one-third, of the genes induced following inoculation with Xoc, Xoo, or either strain are predicted targets of those TAL effectors; and within these predictions multiple targets per TAL effector as well as multiple TAL effectors per target were observed.

### Experimentation verifies 19 targets for *X. oryzae* pv. oryzicola BLS256 TAL effectors

The next step was to determine which predicted TAL effector targets are real targets. Because several *S* genes for bacterial blight of rice have been characterized and all are TAL effector targets, while no *S* genes have yet been identified for bacterial leaf streak and the roles of TAL effectors in this disease have not been explored, we focused on the 44 TAL effector-target pairs predicted for Xoc (Table 1, taking Tal6 and *Os12g42970*, with its two Tal6 EBEs, as one pair). To identify real targets, we used both TAL effector loss of function and gain of function assays to test TAL effector dependence of expression. First we generated a library of Xoc TAL effector mutant strains by marker exchange mutagenesis. By mapping the mutation in several strains, we identified loss of function derivatives for all but one (Tal2a) of the TAL effectors for which we had predicted a target. And, we cloned each of the TAL effectors into a broad host range plasmid for complementation and heterologous expression (gain of function). Then we assessed by RT-PCR whether any TAL effector mutant strain failed to activate any of the corresponding predicted targets of that TAL effector, and for any that did, whether the cloned effector specifically complemented the mutation to restore activation. In parallel, we expressed each TAL effector in strain EB08 of the soybean pathogen *X. axonopodis* pv. glycines (Xag) [41], which neither causes symptoms nor elicits a hypersensitive reaction when inoculated to rice (cv. Nipponbare), and we determined whether the transformants specifically activated corresponding targets.

**Table 1:** Predicted *X. oryzae* pv. oryzicola BL256 TAL effector targets in rice (cv. Nipponbare) induced during infection and results of verification experiments.^a^

TAL effector	Target Locus ID^b^	Probe set ID(s)	Fold change 2–96 h Xoc^c^	Fold Change Mock-Xoc 96 h^d^	*q* (Mock-Xoc)^e^	EBE Score^f^	EBE rel. score^g^	EBE rank^h^	EBE to TLS^i^	EBE to TXS^j^	EBE to TATA box^k^	EBE to Y patch^l^	Induced by		Description
													*tal* gene knockout strain of Xoc^l^	Xag expressing the *tal* gene^m^	
Tal4a	01g27210	Os.7911.1.S1_at	1.63	1.66	1.8E-01	29.22	2.85	341	253	143	−50	none	+	−	Glutathione S-transferase, putative, expressed
Tal6	01g31220	Os.6438.1.S1_a_at	1.48	1.53	8.0E-02	18.75	2.38	685	157	152	none	33	−	+	Expressed protein
		Os.6438.2.S1_x_at	1.48	1.52	1.1E-02										
Tal2d	01g51040	Os.53457.1.S1_at	2.30	2.23	1.9E-01	14.32	2.19	324	527	none	−328	0	+	−	Transmembrane protein 16K, putative, expressed
Tal9b	01g51040	Os.53457.1.S1_at	2.30	2.23	1.9E-01	14.07	2.81	275	18	0	−299	none	−	+	Transmembrane protein 16K, putative, expressed
Tal2g	01g52130	Os.41841.1.S1_at	13.00	9.59	1.3E-06	13.94	1.97	77	427	58	28	none	−	+	Sulfate transporter, putative, expressed
Tal3b	01g53220	Os.35681.1.S1_at	3.50	4.12	2.2E-06	17.72	2.92	611	146	−5	−137	none	nd	nd	HSF-type DNA-binding domain containing protein, expressed
Tal6	02g14770	Os.2450.1.S1_a_at	1.88	1.77	1.3E-02	18.48	2.35	569	92	48	−70	−37	+	−	Phosphoenolpyruvate carboxylase, putative, expressed
		Os.2450.3.S1_x_at	1.85	1.55	6.8E-02										
Tal11a	02g15290	Os.56119.1.S1_at	1.72	4.93	4.1E-07	20.15	3.18	582	422	none	−288	none	+	−	VQ domain containing protein, putative, expressed
Tal5a	02g15290	Os.56119.1.S1_at	1.72	4.93	4.1E-07	23.32	1.88	107	148	30	−3	−180	−	+	VQ domain containing protein, putative, expressed
Tal7	02g15710	OsAffx.2629.1.S1_at	5.70	5.52	8.4E-02	15.75	1.94	265	951	none	150	434	nd	nd	Plastocyanin-like domain containing protein, putative, expressed
Tal3b	02g34970	Os.47735.1.S1_at	9.07	5.31	4.0E-07	15.33	2.53	75	110	29	−282	none	−	+	No apical meristem protein, putative, expressed
Tal2a	02g43760	Os.1349.1.S1_at	1.25	1.45	1.7E-03	15.87	1.75	21	521	none	−334	−5	nd	+	Ubiquitin carboxyl-terminal hydrolase, family 1, putative, expressed
		OsAffx.2950.1.S1_s_at	1.23	1.32	5.3E-03										
Tal7	02g43760	Os.1349.1.S1_at	1.25	1.45	1.7E-03	16.45	2.03	547	628	341	17	117	+	−	Ubiquitin carboxyl-terminal hydrolase, family 1, putative, expressed
		OsAffx.2950.1.S1_s_at	1.23	1.32	5.3E-03										
Tal3c	02g47660	Os.7751.1.S1_at	2.25	2.24	1.9E-03	10.93	1.92	53	140	−63	−98	none	−	+	Basic helix-loop-helix, putative, expressed
Tal4c	02g47660	Os.7751.1.S1_at	2.25	2.24	1.9E-03	24.73	3.01	434	367	310	−25	none	+	−	Basic helix-loop-helix, putative, expressed
Tal2c	03g03034	Os.10510.1.S1_at	1.49	3.11	1.1E-02	19.55	1.83	0	142	114	−779	6	−	+	Flavonol synthase/flavanone 3-hydroxylase, putative, expressed
		Os.53217.1.S1_x_at	1.26	2.83	6.8E-02										
Tal3b	03g03034	Os.10510.1.S1_at	1.49	3.11	1.1E-02	16.73	2.76	258	759	567	none	none	+	−	Flavonol synthase/flavanone 3-hydroxylase, putative, expressed
		Os.53217.1.S1_x_at	1.26	2.83	6.8E-02										
Tal11a	03g05370	OsAffx.24978.1.S1_at	10.98	13.02	5.2E-04	17.39	2.74	307	798	331	526	none	+	−	Expressed protein
Tal3c	03g07540	OsAffx.3165.1.S1_at	6.33	3.84	3.6E-02	12.33	2.17	350	248	99	−625	none	−	+	bHLH family protein, putative, expressed
Tal7	03g25490	Os.34992.2.S1_at	2.10	2.02	3.8E-05	16.32	2.01	494	199	30	−363	9	+	−	Cytochrome P450 72A1, putative, expressed
Tal4a	03g37840	Os.20541.1.S1_at	2.24	1.96	2.2E-04	15.58	1.52	0	362	151	−3	none	−	+	Potassium transporter, putative, expressed
Tal2d	04g49194	Os.17316.1.S1_at	22.42	10.49	3.9E-07	8.22	1.26	0	101	26	−715	none	−	+	Naringenin,2-oxoglutarate 3-dioxygenase, putative, expressed
Tal3a	05g12450	OsAffx.26856.1.S1_at	1.81	1.34	2.3E-01	16.07	2.01	294	446	315	none	none	+	−	Hydroquinone glucosyltransferase, putative, expressed
Tal3b	05g27590	Os.57186.1.S1_at	2.40	4.42	3.4E-08	11.40	1.88	2	103	33	−1	none	−	+	Wound-induced protein WI12, putative, expressed
Tal11b	06g14750	OsAffx.15432.1.S1_at	1.29	1.30	2.0E-01	12.44	2.77	129	313	195	160	−15	+	−	Phosphatidylinositol-4-phosphate 5-Kinase family protein, putative, expressed
Tal1c	06g14750	OsAffx.15432.1.S1_at	1.29	1.30	2.0E-01	12.00	2.44	256	178	47	none	none	+	−	Phosphatidylinositol-4-phosphate 5-Kinase family protein, putative, expressed
Tal2a	06g14750	OsAffx.15432.1.S1_at	1.29	1.30	2.0E-01	17.59	1.94	88	79	−22	−618	−33	nd	−	Phosphatidylinositol-4-phosphate 5-Kinase family protein, putative, expressed
Tal4c	06g37080	Os.16282.1.A1_at	5.54	7.15	2.7E-10	14.64	1.78	0	150	39	−1	none	−	+	L-ascorbate oxidase precursor, putative, expressed
		OsAffx.15788.1.S1_at	11.84	9.94	6.3E-09										
Tal8	06g37080	Os.16282.1.A1_at	5.54	7.15	2.7E-10	19.92	2.32	605	661	560	−36	548	+	−	L-ascorbate oxidase precursor, putative, expressed
		OsAffx.15788.1.S1_at	11.84	9.94	6.3E-09										
Tal2g	06g46500	Os.49496.1.S1_at	6.40	6.88	4.3E-08	14.27	2.01	117	89	59	−489	−47	−	+	Monocopper oxidase, putative, expressed
Tal11a	06g47950	OsAffx.15977.1.S1_s_at	1.78	1.67	2.8E-02	16.20	2.55	19	527	none	−328	0	nd	nd	Tetratricopeptide-like helical, putative, expressed
Tal1c	07g06970	Os.49794.1.S1_at	2.95	2.27	1.3E-02	5.97	1.22	0	216	24	none	none	−	+	HEN1, putative, expressed
Tal3a	07g06970	Os.49794.1.S1_at	2.95	2.27	1.3E-02	16.21	2.03	354	930	815	444	none	+	−	HEN1, putative, expressed
Tal4c	07g29750	Os.46631.1.S1_x_at	6.69	5.05	3.4E-07	25.09	3.05	557	233	30	−5	none	+	−	Glycosyl hydrolases family 16, putative, expressed
Tal4b	07g34510	Os.51294.1.S1_at	0.95	1.00	2.8E-01	8.88	1.63	33	302	151	−425	none	nd	nd	Retrotransposon protein, putative, unclassified, expressed
Tal3b	07g36430	Os.31021.1.S1_at	2.53	2.40	2.6E-02	15.78	2.6	108	117	31	−4	none	−	+	Expressed protein
Tal6	07g47790	Os.8920.1.S1_at	4.16	8.41	3.6E-02	13.38	1.7	8	798	610	−192	694	+	−	AP2 domain containing protein, expressed
Tal4a	09g20220	Os.4759.1.S1_at	2.17	2.38	4.9E-02	28.93	2.83	280	170	139	−751	34	+	−	Glutathione S-transferase, putative, expressed
Tal2d	09g23560	Os.5983.1.S1_at	2.19	5.02	2.8E-01	14.19	2.17	288	525	none	none	93	nd	nd	Dehydrogenase, putative, expressed
Tal6	09g29100	Os.18607.1.S1_at	1.64	1.97	3.6E-02	17.00	2.13	167	0	0	0	0	−	+	Cyclin, putative, expressed
Tal4b	09g32100	Os.16365.1.S1_at	3.34	2.45	8.0E-03	8.15	1.5	16	270	84	21	none	−	+	Expressed protein
Tal9a	11g01480	Os.18448.1.S1_s_at	5.42	3.94	8.2E-06	19.71	2.56	206	776	621	365	none	+	−	MYB family transcription factor, putative, expressed
		OsAffx.30765.1.S1_at	5.74	4.10	5.4E-06										
Tal9a	12g01490	Os.18448.1.S1_at	5.21	3.92	2.6E-05	19.71	2.56	205	302	191	151	none	+	−	MYB family transcription factor, putative, expressed
Tal6	12g42970	Os.11382.1.S1_at	2.31	1.65	2.2E-04	16.84	2.14	139	132	30	−565	12	−	+	GATA zinc finger domain containing protein, expressed
Tal6	12g42970	Os.11382.1.S1_at	2.31	1.65	2.2E-04	18.27	2.32	411	107	5	−590	−13	−	+	GATA zinc finger domain containing protein, expressed

a Expression values are from the GeneChip expression experiment; see Materials and Methods.

b Prefix “LOC_Os” is omitted.

c Fold change in transcript abundance in leaves at 96 h relative to 2 h after inoculation with *X. oryzae* pv. oryzicola BLS256 (Xoc).

d Fold change in transcript abundance at 96 h in Xoc-inoculated leaves relative to mock-inoculated leaves.

e Calculated for the comparison of transcript abundance in Xoc vs. mock inoculated leaves across all time points.

f Score is according to Doyle et al. [32] except that new RVDs ‘SN’ and ‘YG’, present in Tal2g were assigned nucleotide association frequencies of ‘NN’ and ‘NG’, respectively (see text).

g EBE relative score, ratio of the observed EBE score to the best possible score for the TAL effector [32].

h EBE rank among the single best scoring sites for the TAL effector in each rice promoter [32].

i Distance in bases from the 5′ end of the EBE to the translational start site (TLS) of the target locus; a positive value indicates a location downstream of the EBE.

j Distance in bases from the 5′ end of the EBE to the transcriptional start site (TXS) based on cDNA evidence in the Rice Genome Annotation Project Release 7 (http://rice.plantbiology.msu.edu/); a positive value indicates a location downstream of the EBE; none, cDNA evidence of TXS missing.

k Distance in bases from the 5′ end of the EBE to the nearest identified putative TATA box; a positive value indicates a location downstream of the EBE; none, putative TATA box not present.

l Distance in bases from the 5′ end of the EBE to the nearest identified putative Y patch; a positive value indicates a location downstream of the EBE; none, putative Y patch not present.

m Results of RT-PCR 48 h after inoculation, relative to a negative control inoculation (see Supplemental Figure S1); Xoc, *X. oryzae* pv. oryzicola BLS256; Xag, *X. axonopodis* pv glycines EB08; +, induced; −, not induced; nd, transcript not detected by RT-PCR (in each case, amplification by standard PCR from genomic DNA as template was confirmed).

The results verified 19 of the 44 predicted Xoc TAL-effector targets as real (Table 1 and Figure S1; the Tal2a target was verified only by the gain of function experiment). Another 20 were shown not to be activated by the corresponding TAL effector and are hereafter referred to as falsely predicted targets. The remaining five could not be tested because transcript was not detected by RT-PCR, despite induction according to the GeneChip expression data. Interestingly, multiple predicted targets were verified for some TAL effectors, however, for each of the eight genes predicted to be targeted by multiple TAL effectors, only activation by one of those TAL effectors was verified.

### Most *X. oryzae* pv. oryzicola BLS256 TAL effectors have no significant role in virulence

**Figure 4:**
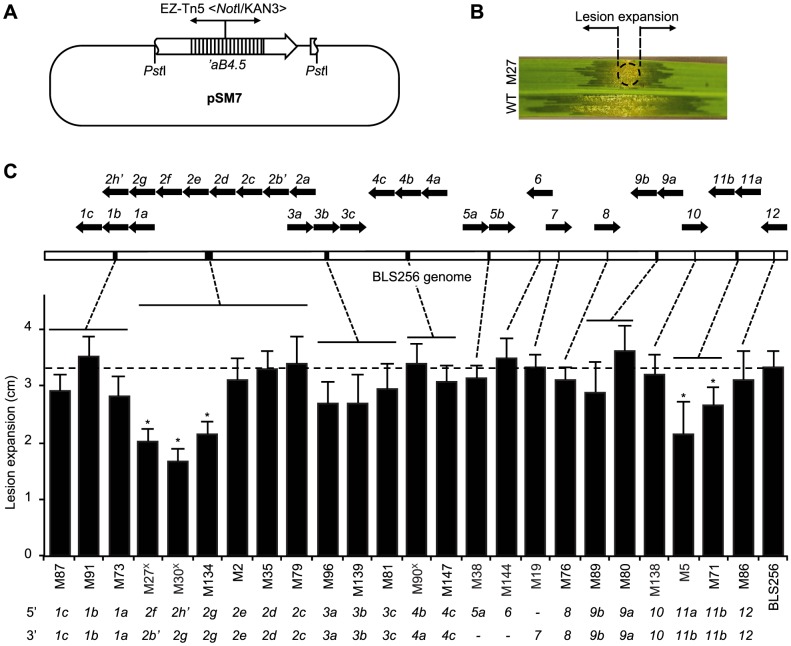
Virulence of *X. oryzae* pv. oryzicola BLS256 *tal* gene knockout strains. (A) Suicide plasmid pSM7 (Table S8) used for *tal* gene knockouts by homologous recombination in BLS256. pSM7 harbors a 4.5-kb *Pst*I fragment containing all but the first 80 bp of the ORF of *tal* gene *aB4.5* [12] with an insertion of the EZ-Tn5 <*Not*I/KAN-3> transposon (Epicentre) in repeat 9, in pBluescript II KS(+) (Agilent), which does not replicate in *Xanthomonas*. The transposon provides kanamycin resistance for selection. Because the *tal* ORF is truncated at the 5′ end, either a single or double recombination that retains the transposon results in a *tal* gene knockout. Double recombination can knock out clustered *tal* genes. The 4.5 kb *Pst*I fragment also includes the first 85 bp of the *avrXa10 tal* gene downstream of *ab4.5*, which might increase the likelihood of complex recombination. (B) Virulence assay used to characterize knockout strains. Suspensions of mutant and wild-type cells are inoculated side by side via leaf infiltration of 4-week old plants using a needless syringe, and expansion of lesions from the inoculation site (circle), as shown for mutant M27 in this example, is measured after 7 days. (C) Virulence of knockout strains and mapped endpoints of integrations. Only strains with single integrations as determined by Southern blot (not shown) were further characterized. Integration endpoints were mapped by PCR amplification of flanking DNA, using transposon-specific and *tal* gene conserved end specific primers, and sequencing. BLS256 *tal* gene polymorphisms in most cases enabled unambiguous mapping. Virulence results are plotted left to right in the histogram by integration location, indicated by dashed lines pointing to a linearized representation of the genome, above, with individual *tal* gene clusters indicated by black bars and magnified at top to show gene content and orientation using block arrows. An apostrophe denotes a pseudogene. At bottom, integration endpoints for each mutant strain are given, by *tal* gene. A dash means the endpoint could not be unambiguously determined. A superscript “X” after the mutant strain designation denotes an apparent complex recombination, suggested by the 5′ endpoint mapping downstream of the 3′ endpoint. In the histogram, an asterisk indicates significantly reduced virulence (*p*<0.01, N = 10) relative to wild type. Assays were repeated at least three times with consistent results.

Having identified 19 targets of Xoc TAL effectors, the next challenge was to ascertain whether any are *S* genes for bacterial leaf streak. Barring redundancy, *i.e.*, targeting of the same *S* gene by multiple TAL effectors, which our verification experiments excluded for each target tested, loss of a TAL effector that activates an important *S* gene should by definition result in a reduction of virulence. We therefore first quantified the virulence of each of several mutant strains of Xoc to identify such TAL effectors, using a lesion length assay (Figure 4). Collectively, the mutants account for all 26 Xoc TAL effectors except Tal2a, for which a mutant was not isolated. Assayed on rice cv. Nipponbare plants, only mutations that map on at least one side to the 3′ end of the *tal2* cluster, i.e., involving *tal2f* or *tal2g*, or that map to the *tal11* cluster, which contains *tal11a* and *tal11b*, were associated with significantly reduced virulence, 49–64% and 64–79%, respectively. Thus, most of the Xoc TAL effectors, in the context of the Nipponbare host genotype, appear not to make any non-redundant, major contributions to virulence. Interestingly, this includes the TAL effectors that activate genes induced in common by Xoc and Xoo, Tal1c, Tal2c, and Tal5a (Table 1, Table S3, and Table S7).

### Tal2g is a major virulence factor of *Xanthomonas oryzae* pv. oryzicola BLS256

**Figure 5:**
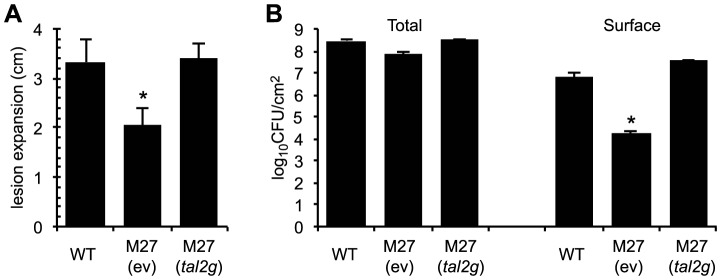
Virulence contribution of *X. oryzae* pv. oryzicola BLS256 TAL effector Tal2g. (A) Lengths of lesions caused by *X. oryzae* pv. oryzicola BLS256 (WT), the *tal2g* knockout derivative M27 carrying an empty plasmid vector (ev), and M27 carrying the vector with the cloned *tal2g* gene, measured as in Figure 4, but at 10 days after infiltration. The asterisk indicates a significant difference relative to WT (*p*<0.01). Error bars represent standard deviation (N≥10). (B) Total and surface (exudate) bacterial populations of leaves seven days after inoculation with the strains in panel A. The asterisk indicates a significant difference relative to WT (*p*<0.01). Error bars represent standard deviation (N≥6). Experiments were repeated three times with consistent results.

Of the few Xoc TAL effectors pinpointed by the mutational analysis as possible virulence factors that might lead us to one or more *S* genes (Tal2f, Tal2g, Tal11a, and Tal11b), we had verified targets only for Tal2g (Table 1). From the code- and GeneChip expression-based analysis, Tal2f had no predicted targets, and two of the three predicted targets of Tal11a and the sole predicted target of Tal11b were shown not to be actual targets by the loss- and gain-of-function RT-PCR experiments (Table 1). So, we focused on Tal2g. Of the three mutant strains in which the mutation endpoints map within or flanking Tal2g (Figure 4: M27, M30, and M134), we chose mutant M27 for further characterization. In M27, the marker exchange endpoints suggest a complex recombination, with a disrupted *tal2f* on the 5′ end and a disrupted *tal2b′*, a pseudogene that resides 5′ of *tal2f* in the native chromosome, on the 3′ end. Because the apparent complex recombination might have affected several genes in the cluster, we assayed each *tal2* gene (*tal2a*, -*c*, -*d*, -*e*, -*f*, and -*g*), individually on a plasmid for the ability to complement M27. Only *tal2g* restored virulence to M27 in the lesion length assay, and it did so fully, confirming Tal2g as the sole virulence factor among the TAL effectors whose expression is disrupted in this mutant (Figure 5A). The marker exchange endpoints in M27 could be explained by a double crossover between *tal2b′* and *tal2g*, concurrent with the marker exchange crossovers, that positioned *tal2b′* sequences at the 3′ endpoint of the exchange, with the 5′ end in *tal2f*, disrupting *tal2g* but not affecting *tal2c*, *tal2d*, or *tal2e*. Consistent with this, the verified targets of Tal2c and Tal2d (*Os03g03034* and *Os04g49194*) are induced by M27 (Figure S2).

Curiously, the total population of M27 isolated from leaf homogenates at seven days after inoculation was not significantly different from that of the wild type (Figure 5B). However, we observed less bacterial exudate on the surface of M27-inoculated leaves than on leaves inoculated with wild type (see Figure 4B). When surface bacteria were isolated and quantified (see Materials and Methods), M27 indeed showed nearly a 400-fold reduction relative to the wild type, and Tal2g on a plasmid fully restored wild-type levels of exudate (Figure 5B). Thus, Tal2g is a major virulence factor in bacterial leaf streak that functions both in lesion expansion and exudation of bacteria to the leaf surface.

### A sulfate transporter gene targeted by Tal2g is a major susceptibility gene for bacterial leaf streak

**Figure 6:**
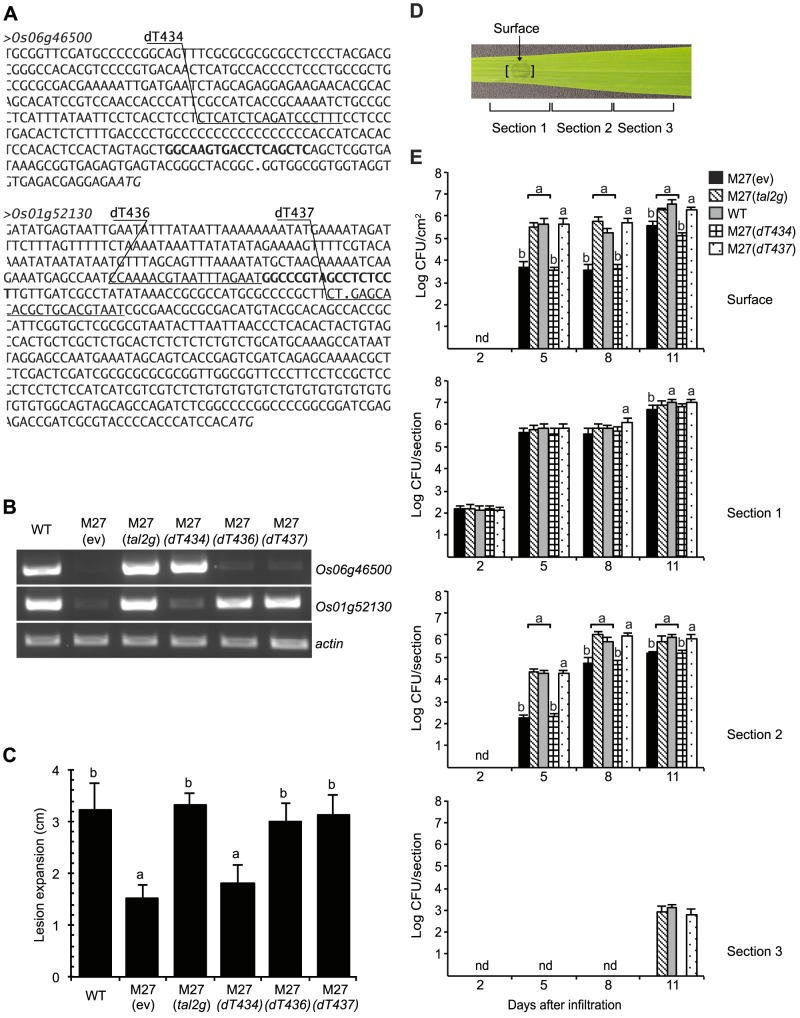
Determination of *Os01g52130* as the relevant target of Tal2g using designer TAL effectors. (A) DNA sequence of the promoter regions of Tal2g induced genes *Os06g46500* and *Os01g52130* in rice cv. Nipponbare. The effector binding elements (EBEs) for Tal2g are in bold. The EBEs for designer TAL effectors dT434 targeting *Os06g46500* and dT436 and dT437 targeting *Os01g52130* are underlined and labeled above. Periods indicate transcriptional start sites and italics indicate translational start sites, per the Rice Genome Annotation Project (Release 7, http://rice.plantbiology.msu.edu). (B) Activation of *Os06g46500* and *Os1g52130* by Tal2g, and specific activation respectively of *Os06g46500* and *Os01g52130* by dT434, and dT436 or dT437. Shown are the results of RT-PCR amplification from leaf RNA isolated 48 h after inoculation by infiltration with *X. oryzae* pv. oryzicola BLS256 (WT), the *tal2g* knockout derivative M27 carrying an empty plasmid vector (ev), M27 carrying the vector with the cloned *tal2g* gene, or M27 carrying the vector with coding sequences for dT436, dT436, or dT437 as indicated. The actin gene *Os04g57210* was used as a reference for relative transcript abundance across samples. (C) Rescue of the virulence defect of M27 by dT436 or dT437 but not dT434 in the lesion length assay. Lesion lengths were measured as in Figure 4, 10 days after inoculation with the indicated strains. Values labeled with the same letter are not significantly different and those labeled with different letters are (Student's *t*-test, p<0.01). Error bars represent standard deviation (N≥10). Experiments were repeated twice with consistent results. (D) A rice (cv. Nipponbare) leaf showing bacterial leaf streak symptoms two days after inoculation with a suspension of WT cells at an OD_600_ of 0.5 (approximately 1×10^8^ CFU/ml) by infiltration using a needleless syringe over a 4 mm diameter leaf area, and labeled to indicate the site of inoculation, at which surface bacterial populations were quantified, and the three 12 mm long leaf sections in which total bacterial populations were quantified, as presented in panel E. (E) Restoration of the surface population and the total population spread of M27 to wild-type levels by dTAL437 but not dTAL434. Populations were quantified at 2, 5, 8 and 11 days after inoculation. Results are the means and standard deviations of samples from three leaves; nd, not detected. At each time point (not across time points), values labeled with the same letter are not significantly different, and those labeled with different letters are (Student's *t*-test *p*<0.0001).

The two verified targets of Tal2g, *Os06g46500*, encoding a predicted monocopper oxidase, and *Os01g52130*, encoding a predicted sulfate transporter, *OsSULTR3;6* [42], are among the most significantly induced genes in the GeneChip expression dataset (Table S1). To test whether either is a biologically relevant target, i.e., an *S* gene, we engineered designer TAL effectors (dTALEs) to specifically activate each target individually, and we tested the ability of these dTALEs to restore virulence to M27 (Figure 6). Assayed by RT-PCR, in syringe infiltrated leaves dTALE dT434 expressed in M27 specifically induced the monocopper oxidase gene, and dTALEs dT436 or dT437 induced *OsSULTR3;6*, each similarly to wild type and to M27 expressing Tal2g (Figure 6B). In the lesion length assay, dT436 and dT437 each restored full virulence to M27, whereas dT434 made no significant difference (Figure 6C). When surface bacterial populations were quantified over time at the inoculation site, and spread of bacteria over time was measured by quantifying total populations in contiguous leaf segments at and extending from the inoculation site, M27 expressing dT437 and M27 expressing Tal2g behaved the same as the wild type, whereas M27 expressing dT434 showed a reduction in surface population and slowed population spread equivalent to M27 carrying the empty vector (Figure 6D and Figure 6E). Scanning the rice promoterome for candidate EBEs as in our original search for potential Xoc and Xoo TAL effector targets, we found no overlap between candidate off-targets of dT436 and dT437, or between off-targets of either with genes harboring a potential Tal2g EBE. Together, the data therefore indicate that *OsSULTR3;6* is the relevant Tal2g target and a major *S* gene for bacterial leaf streak.

### Functional characterization of Tal2g EBEs and similarly scored sequences supports presumed specificities of new RVDs ‘SN’ and ‘YG’

As described above, in our search for TAL effector targets, we used specificity values of ‘NN’ and ‘NG’ for the ‘SN’ and ‘YG’ RVDs that are found in Tal2g. As might be expected, the list of candidate Tal2g EBEs generated using these values differed from a second list we generated in parallel using the default, wild card values. Specifically, in the list generated using the default values for ‘SN’ and ‘YG’, hereafter referred to as the default scoring list, the verified Tal2g target *Os06g46500* did not make the cutoff (Materials and Methods) to be considered a candidate (indeed no sequence from any Xoc-induced gene beside *OsSULTR3;6* scored well enough in this list to be considered a candidate), indicating that substituting the RVD specificity values allowed us to capture an otherwise false negative.

**Figure 7:**
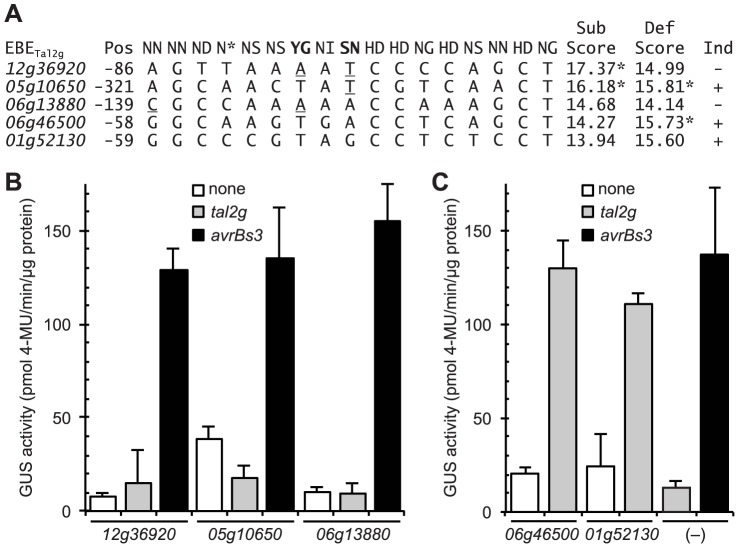
Functional characterization of selected rice promoter sequences similar to the verified Tal2g EBEs. (A) Alignment of selected rice promoter sequences (from loci *Os06g13880*, *Os12g36920*, and *Os05g10650*; see text) and EBEs from the verified Tal2g targets *Os01g52130* (*OsSULTR3;*6) and *Os06g46500* with the corresponding sequence of repeat variable diresidues (RVD) of Tal2g. Position (Pos) is that of the 5′ end relative to the annotated transcriptional start site. Rare RVDs ‘YG’ and ‘SN’ of Tal2g are in bold. Scores were calculated according to [32], either substituting the nucleotide association frequencies of common RVDs ‘NN’ and ‘NG’ for the new RVDs ‘SN’ and ‘YG’ (“Sub Scores”) or using the default wild card specificity values for the new RVDs (“Def Scores”). An asterisk indicates that the score is outside the cutoff to be considered a candidate EBE for Tal2g, calculated independently for each scoring method. Nucleotide mismatches to the new RVDs using the substituted specificities are underlined, as is a (5′) mismatch in the *06g13880* sequence to the first RVD (‘NN’) of Tal2g. Whether a gene is induced (Ind) upon infection by *Xanthomonas oryzae* pv. oryzicola BLS256 is indicated by a plus or minus sign at right. (B) Activity of the selected sequences in an *Agrobacterium*-mediated transient transformation based reporter assay in *Nicotiana benthamiana* leaves [40]. In this assay, a TAL effector gene (none, *tal2g*, or *avrBs3*) driven by the 35S promoter is introduced together with the GUS gene under the control of a minimal promoter from the pepper *Bs3* gene, with the test sequence inserted slightly upstream of the native EBE for AvrBs3 (AvrBs3 is the TAL effector from the pepper pathogen *X. euvesicatoria* that activates *Bs3* upon infection). The inserted sequences are indicated by locus ID on the X axis; “(–)” indicates the minimal *Bs3* promoter with only the AvrBs3 EBE and no added sequence. Error bars represent standard deviation (N = 3). Experiments were repeated twice with consistent results. (C) Activity and specificity of the EBEs from the two verified targets of Tal2g, as in panel B.

To further probe the validity of substituting the values, we tested the function of two candidate EBEs from the default scoring list that each scored better (lower; see Materials and Methods) than the (default-scored) EBEs in the two verified targets, but that displayed a mismatch to one or each of the two new RVDs in Tal2g based on the presumed specificities of those RVDs (Figure 7A). Though not induced by Xoc, both of the corresponding genes, *Os06g13880* and *Os12g36920*, are induced by Xoo (Table S2), indicating that they are euchromatic. Also, the default-scored candidate EBEs, at 139 bp and 86 bp upstream of the respective annotated transcriptional start sites, are each within the range of locations displayed by the EBEs in all the targets verified in this study (152 bp or less; Table 1), so failure to be induced by Xoc likely does not relate to suboptimal EBE localization. We also chose to test a third sequence with a mismatch to one of the new RVDs, that scored just above the cutoff in the default scoring list (Figure 7A) and was therefore not considered a candidate, but was nonetheless in the promoter of an Xoc-induced gene *Os05g10650* (Table S1), and therefore a potential false negative in that list. To test the function of the three sequences, we used a transient, *Agrobacterium*-mediated, TAL effector-driven reporter gene expression assay in *Nicotiana benthamiana* [40]. None of the sequences, inserted into a 343 bp fragment of the pepper *Bs3* promoter just upstream of the native EBE for the cognate TAL effector AvrBs3 [25], rendered the reporter responsive to Tal2g (Figure 7B). In contrast, the EBEs from the verified targets of Tal2g resulted in strong and specific induction of the reporter by Tal2g similar to induction of the unamended reporter by AvrBs3 (Figure 7C).

Thus, in addition to capturing the verified target *Os06g46500* as a candidate, the substituted scoring correctly classifies the *Os12g36920* and *Os05g10650* sequences as non-candidates (scored above the cutoff). The substituted scoring scores the *Os06g13880* sequence as worse than the EBEs of the two verified targets, consistent with its lack of activity, but still calls it a candidate. This incongruity might be explained by the observation that the *Os06g13880* sequence displays a mismatch to the first RVD of Tal2g (Figure 7A), and mismatches at the 5′ end and especially at the first position have been shown to more strongly negatively affect activity than mismatches elsewhere [43] a phenomenon not accounted for by the scoring function. Taken together, the observations overall support the assignment of the common RVD specificities for those of the new cognate RVDs, in agreement with the inference from published structural data discussed earlier.

### Target upregulation by TAL effectors on average is moderate

**Figure 8:**
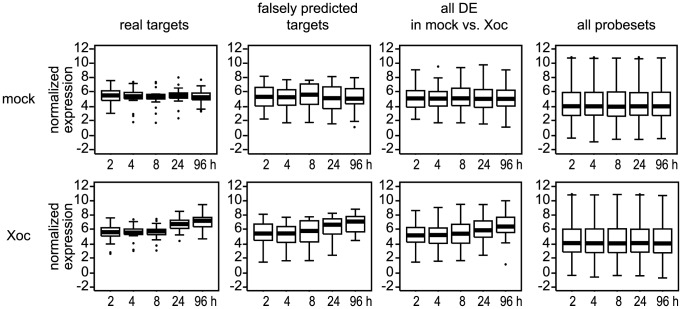
Expression levels of probesets associated with *X. oryzae* pv. oryzicola BLS256 (Xoc) TAL effector targets relative to other probesets. Individual box plots show average normalized expression values over time for probesets associated with verified (real) Xoc TAL effector targets, probesets associated with genes predicted but shown not to be targeted by an Xoc TAL effector (falsely predicted targets), all probesets differentially expressed (DE) in the mock vs. Xoc comparison at *q*≤0.3, or all probesets on the chip. The top row of plots shows data from mock-inoculated plants and the bottom row data from plants inoculated with Xoc. For each plot, the central bar indicates the median value and the top and bottom of the box indicate the 75^th^ percentile and the 25^th^ percentile, respectively. Whiskers indicate the most extreme data points above and below the median that are not outliers, calculated as ≤1.5*(75^th^ percentile – 25^th^ percentile) above the 75^th^ percentile or below the 25^th^ percentile. Outliers are plotted individually. Boxplots were made using the ‘boxplot()’ function of the statistical software package R (www.r-project.org).

Returning to our list of 19 new, verified TAL effector-target pairs, we next sought to determine whether the expression patterns of the targets might reveal general characteristics of TAL effector-driven gene expression. Using the normalized (log_2_ transformed) GeneChip expression data, we began by comparing the average transcript levels of the targets at two hours after inoculation in mock- or Xoc-inoculated plants to expression levels of 1) the 20 falsely predicted targets, 2) all genes differentially expressed (DE) in the Xoc vs. mock comparison, and 3) all genes represented on the array (Figure 8). This average basal expression level of the targets was nearly identical in mock- and Xoc-inoculated plants, similar to that of the falsely predicted targets, slightly higher than that of all genes DE in the mock vs. Xoc comparison, and markedly higher than the average expression level for all genes under either condition at any time point (4.4). Indeed, the majority (14 of 19) of the targets showed basal levels (two hours after inoculation with Xoc) higher than that average (Table S7; for the analyses presented here and throughout this section, genes represented by two probesets in any table were assigned the average values of those probesets). The target with the highest normalized basal expression level was *Os03g37840* targeted by Tal4a, at 7.6, approximately 1.7 times the genome-wide average at that time for either Xoc- or mock-inoculated plants.

We next examined expression at two hours after Xoc inoculation relative to expression at 96 hours after that treatment. The average fold induction (Table 1 and Table S7) of the targets (calculated as 2^average(X-Y)^, where fold induction of a gene is defined as 2^(X-Y)^ for the difference between normalized expression values X and Y; see Materials and Methods) was 3.3, compared to an average of 2.7 for the falsely predicted targets (Table 1 and Table S7) and 2.6 for all the genes DE in the Xoc vs. mock comparison (Table S1 and Table S3). Compared to the average for all 19 targets, induction of 11 of the 14 targets with higher than average basal expression levels was moderate, from the overall minimum of 1.2-fold, exhibited by the Tal2a target *Os02g43760*, to 3.3-fold, whereas the five targets basally expressed at or below the average for the genome were induced 1.6- to 9.1-fold. The remaining three targets, which were expressed at higher than average basal levels, varied in their induction from 6.4- to the overall high of 22.4-fold exhibited by the Tal2d target *Os04g49194*. This latter value was second only to the 34.2-fold induction of *Os01g40290* (Table S1), a gene not predicted to be an Xoc TAL effector target. The normalized expression value for the Tal2d target *Os04g49194* at 96 hours after Xoc inoculation, 9.4, was also near the maximum across the genome for that time point and treatment, 10.7 (*Os11g47970*, probeset Os.11573.2.A2_a_at). Right behind was the sulfate transporter *S* gene *Os01g52130* targeted by Tal2g, exhibiting induction of 13.0-fold to an expression level of 9.1. Overall, though there was not a perfect inverse correlation between basal expression level and fold-induction, expression levels of all targets at 96 hours after Xoc inoculation varied relatively little, averaging 6.9 (standard deviation, SD,1.3), suggesting that regardless of initial target expression level, TAL effectors may generally induce genes to a similar final level.

Extending the analysis to the four known Xoo TAL effector-target pairs represented in our data (Table S7), we found that the average basal expression (*i.e.*, two hours following *Xoo* infection) was 5.4 (SD 0.6), nearly identical to the average basal expression of *Xoc* TAL effector targets (5.2 with SD 1.3). One of the *Xoo* TAL effector targets (*Os07g06970* targeted by Tal9a, also targeted by Tal1c of Xoc) was expressed basally at near genome-average levels. It was moderately induced, 5.0-fold, by 96 hours after Xoo inoculation. The other three, like the majority of the Xoc TAL effector targets, were each basally expressed at higher than average levels. Two of these, *Os01g73890* (*TFIIAγ1*) and *Os09g29820* (*OsTFX1*), targeted by PthXo7 and PthXo6, respectively, also showed relatively low fold induction (3.2- and 2.2-fold, respectively). The overall average fold induction, 4.9, was higher than that of the *Xoc* TAL effector targets, but this number is skewed somewhat by the large change, 17.1-fold, in expression of the third target initially expressed at higher than average levels, *Os08g42350* (*OsSWEET11*) targeted by PthXo1. Despite the small sample size, and with the PthXo1 target as a notable exception, the pattern of expression and fold-induction of the Xoo TAL effector targets overall was similar to that observed for *Xoc* TAL effector targets, tending toward higher than average initial levels and relatively moderate induction.

### EBE features are predictive of real targets

Finally, to better understand targeting and to improve prediction, we asked whether there are features of EBEs in the real targets we identified that distinguish them from those in our falsely predicted targets. Indeed, inspection of the features listed in Table 1 revealed some that appear to be characteristic of EBEs in real targets (we included both Tal6 EBEs in *Os12g42970* in this analysis, for a total of 20 EBEs in real targets). First, on average, EBEs in real targets had lower relative scores. The relative score is the ratio of the actual score for a TAL effector-target alignment to the hypothetical score of that TAL effector aligned with its perfect match target; it allows comparison across TAL effectors, which is otherwise not possible because repeat number and RVD composition affect actual score [32]. The average relative score for EBEs in real targets was 1.98 (range 1.22–2.81), while for falsely predicted targets it was 2.47 (range 1.70–3.18). Second, EBEs in real targets generally ranked more highly in the collection of scores for the TAL effector across all rice promoters than the EBEs in the falsely predicted targets did: 16 of the 20 in real targets ranked in the top 200, with an average rank of 137 across all 20, while 17 of the 20 in falsely predicted targets ranked lower than 200, with an average rank of 347 for all 20. Finally, the maximum distance of an EBE in a real target from the annotated transcriptional start site was 152 bp upstream, with an average of 47 bp upstream (based on 19 that have an annotated TXS, out of the 20 total; range, 152 bp upstream to 63 bp downstream), whereas for the falsely predicted targets, the EBEs were anywhere from 22 bp downstream to 815 bp upstream, with an average distance of 293 bp upstream (based on the 18 with an annotated TXS). Proximity to a TATA box did not appear to correlate independently: nine of the EBEs in real and six of the EBEs in falsely predicted targets are within 100 bp of a TATA box.

To test whether the apparent differences in EBE features could be used to computationally discriminate between real and falsely predicted TAL effector targets and thereby improve future prediction, we took a machine learning approach and trained several Naive Bayes and logistic regression classifiers using combinations of relative score, rank, distance to TXS, and proximity to a TATA box, as well as actual score, distance to translational start site (TLS), and distance to a Y patch, a core promoter motif commonly found in plants [44]. For this analysis, we included also the known Xoo (PXO99^A^) TAL effector targets in Nipponbare, each of which, as noted above, was among our predictions (Table S7). Classifiers were generated using leave-one-out cross validation, a method that determines model parameters using all but one of the EBEs as the training set and then asks whether the resulting classifier correctly calls the remaining EBE. This is repeated with each EBE in turn to optimize the model. Recall, precision, and other metrics are computed based on the number of EBEs classified correctly using this procedure. A Naive Bayes classifier trained on all features achieved the highest recall, capturing 92% of the real targets (Table 2). The precision (percent of positives called that are true positives) of the classifier was 88% (Table 2), and no other classifier had a significantly better area under the receiver operating characteristic curve (AUC; Figure S3), a measure of the tradeoff between recall and precision. Notably, a logistic regression classifier using the distance to transcriptional start site alone achieved a recall almost as high as that achieved using all features, and had a similar AUC (Table 2 and Figure S3).

**Table 2:** Performance of a Naive Bayes classifier trained on all EBE features and of a logistic regression classifier trained on distance to transcriptional start site (TXS) using leave-one-out cross validation.^a^

Features	Accuracy	Precision	Recall	F measure	MCC	AUC
All	.89	.88	.92	.90	.77	.88
Distance to TXS	.87	.88	.88	.88	.73	.87

a See text for features included. Accuracy, precision, and recall are at the maximum F measure obtained by varying the discrimination threshold. Using TP, TN, FP, FN to represent numbers of true positives, true negatives, false positives, and false negatives, respectively, accuracy is 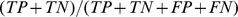, precision is 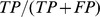, recall is 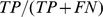, F measure is 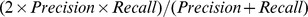, MCC (Matthews correlation coefficient) is 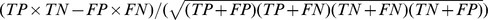, and AUC is the area under the receiver operating characteristic curve, a curve created by plotting TP vs. FP as the discrimination threshold is varied.

## Discussion

In this study we integrated genome-wide expression profiling, computational prediction using the TAL effector-DNA binding code, and functional analyses, and identified a TAL effector target in rice, *OsSULTR3;6*, that plays a major role in susceptibility of this staple crop species to a disease of increasing global importance, bacterial leaf streak of rice. Key to identifying the *S* gene was targeted gene activation using designer TAL effectors. Encoding a predicted sulfate transporter, the gene represents a new class of TAL effector-induced *S* gene, distinct from the handful that has been identified for bacterial blight of rice. Indeed, we discovered that overall, pathogen-induced host transcriptional changes in leaf streak are almost entirely different from those that take place during blight. We found that the T3S-translocated TAL effectors of the leaf streak pathogen are responsible, at a minimum, for nearly a quarter (19/85 genes) of the differential host gene expression during infection that we detected. We identified Tal2g as the major Xoc virulence factor that upregulates *OsSULTR3;6*, and demonstrated that the upregulation of *OsSULTR3;6* contributes specifically to lesion expansion and bacterial exudation. We learned that, on average, TAL effector targets are expressed basally at higher than genome average levels and induced to a moderate extent, though *OsSULTR3;6* and the blight *S* gene *OsSWEET11* were exceptions, as two of the most highly induced genes in our dataset. Finally, the targets we identified and predictions we verified to be false allowed us to generate a Naive Bayes classifier that can be used in the future to identify the strongest candidate TAL effector targets prior to verification experiments, and that may also help optimize targeting with dTALEs. These advances leave the key question about tissue specificity unanswered, and raise other questions, but they open promising new avenues of inquiry. Also, they highlight gaps in our understanding of gene activation by TAL effectors, and point to challenges that remain in code-assisted discovery of TAL effector targets, but they demonstrate nonetheless the power of the approach we used to rapidly dissect interactions between TAL effector-wielding pathogens and their hosts.

### Tissue specificity and the role of TAL effectors

Regarding the basis for the tissue specificity of Xoc relative to Xoo, the markedly distinct patterns of host global gene expression during bacterial leaf streak compared to bacterial blight suggest a role for host gene manipulation by the pathogens. The results of the gene ontology enrichment analysis we carried out on the differentially expressed genes raise the intriguing possibility that Xoc is uniquely able to control redox status, preventing or dampening the defense-associated oxidative burst and or affecting redox-dependent signaling pathways in the mesophyll. In a preliminary experiment to test this possibility, we observed that Xoc-inoculated leaves do show reduced overall H_2_O_2_ content at 96 h after inoculation relative to Xoo- and mock-inoculated plants (Figure S4). The reduction could relate to reduced photosynthesis, as leaves are beginning to exhibit watersoaking by this time, but it could be the direct consequence of Xoc-dependent changes in transcript levels of the redox-modulating genes, as Xoo-infected plants also exhibit watersoaking by 96 hrs, yet are unaltered in their H_2_0_2_ content relative to mock inoculated plants.

In contrast, the abundance of membrane associated and vesicle associated terms unique to the Xoo-induced genes is consistent with an ability to manipulate trafficking pathways that might result in nutrient export from xylem parenchyma cells into the nutrient-poor xylem, an ability Xoc may lack. This possibility aligns with the presumed role of the blight *S* gene *OsSWEET11* as a sucrose exporter.

The extent to which TAL effectors account for the genome-wide differences in gene expression is uncertain. We observed previously that TAL effectors in Xoc and Xoo diversified subsequent to or in concert with divergence of the two pathovars [23], so it is tempting to assume a determinative role for TAL effectors in tissue specificity. However, despite our demonstration that 19 out of the 85 genes induced by Xoc are TAL effector targets, the numbers of identified targets, particularly for Xoo, are still too few to draw any conclusions from ontology enrichment analysis of just the targets. But note that targets of TAL effectors from each pathovar include one or more distinct transcription factor or putative transcription factor (bHLH family) genes: the ontology enrichment results just discussed might reflect a pervasive and determinative role of TAL effectors, through both direct and indirect effects on global gene expression patterns.

Genetic manipulation of host cells tailored to the different conditions in the mesophyll apoplast vs. the xylem is a compelling hypothesis, but one might expect some generic manipulation important for colonization both by Xoc and Xoo as well. Curiously, neither of the two genes targeted by TAL effectors from both pathovars, the *OsHen1* RNA methylase gene *Os07g06970* or the VQ domain containing protein gene *Os02g15290*, appears to play an important role in leaf streak, based on the observation that the corresponding TAL effector mutant strains M87 (*tal1c*) and M38 (*tal5a*) were not significantly reduced in virulence (Figure 4). Possible roles of these targets in bacterial blight remain to be tested.

### Virulence contributions of Xoc TAL effectors

Despite the fact that exactly half of the Xoc TAL effectors were found to activate specific targets, most of the Xoc TAL effectors appear not to play a significant role in virulence, raising the question why the pathogen harbors all 26. We screened over 150 pSM7 integrants of Xoc to find that ones showing significantly reduced lesion lengths when inoculated to rice cv. Nipponbare mapped exclusively to *tal* clusters *2* or *11* (Figure 4 shows representative mutants). We narrowed this further to those that affect *tal2g* or the two *tal11* genes. We confirmed the virulence contribution of *tal2g* by complementation, but we did not do the same for the *tal11* mutants, leaving open the possibility even that the reduced virulence of the *tal11a* and *tal11b* mutants was due to ectopic mutation. The lack of a detectable virulence contribution for most Xoc TAL effectors is not unlike observations for Xoo, in which TAL effectors contribute to virulence to different extents, with typically only one or two out of many per strain playing a major role [8], [12]. Three possible reasons for the phenomenon come to mind, none mutually exclusive. First, the non-contributing TAL effectors may be important in host genotypes other than Nipponbare, in which promoter polymorphisms can influence targeting, or in plants at different growth stages from the one we assayed, in which the physiological context might change the gene activation requirements for the development of leaf streak. Second, having many clusters of *tal* genes in the genome, even if most are inconsequential to infection, might provide a selective advantage over time by increasing the likelihood of recombination for adaptation to new host genotypes [45]. Finally, the contributions might be redundant, or subtle, similar to those of non-TAL type III effectors [46]. Though predicted, we confirmed no redundant targeting by Xoc TAL effectors. Rather, the functions of distinct targets could themselves be redundant or epistatic to one another, a scenario that would have escaped detection in our study, but again would provide a pathogen advantage in the face of host genotypic variation. Regarding subtle contributions of individual TAL effectors, they might collectively cause an essential perturbation.

### The role of Tal2g and its relevant target

The importance of Tal2g and the sulfate transporter gene it upregulates for lesion expansion and bacterial exudation is reminiscent of the phenotype associated with TAL effector Avrb6 of the cotton (*Gossypium hirsutum*) pathogen *X. campestris* pv malvacearum. Strains carrying the *avrb6* gene cause larger water-soaked symptoms that correlate with more bacterial release to the leaf surface [6]. It has been proposed that bacterial exit and accumulation onto the leaf surface is advantageous as a means of dissemination, particularly for pathogens like Xoc that do not cause systemic infections [14], [47], [48]. AvrBs3 causes cell hypertrophy that may achieve this by reducing the volume of the apoplast and squeezing bacteria out to the surface, by inducing the pepper cell size regulatory gene *UPA20* [14], [16]. PthA of *X. citri* triggers developmental changes that result in canker formation and rupture, releasing bacteria to the leaf surface [49]. Its target has not been reported. We have seen no evidence of hyperplasia or hypertrophy in available micrographs of Xoc infected rice leaves, nor in electron micrographs we have generated, and sulfate transporters are not known to regulate cell growth, but this possibility should be examined more closely in a future study. We hypothesize though that, as suggested by the effect of Avrb6 (the target of which is also yet to be reported), the enhanced watersoaking conferred by Tal2g upregulation of *OsSULTR3;6* facilitates bacterial egress.

The rice cv. Nipponbare genome encodes 14 sulfate transporter genes phylogenetically divided into five groups [42], [50]. *OsSULTR3;6* belongs to the less well characterized group 3 that includes five additional members. None of the additional members is induced by Xoc (i.e., they are absent from Table S1). A recent report demonstrated a role for the *Arabidopsis* group 3 sulfate transporter *AtSULTR3;1* in pH-dependent sulfate uptake by chloroplasts [51]. The chloroplast is a main site for sulfate reductive assimilation for the synthesis of cysteine, which together with glutathione maintains the antioxidant capacity of the cytosol [52]–[54]. *AtSULTR3;2*, *AtSULTR3;3* and *AtSULTR3;4* also were shown to contribute [51]. In contrast, the last member of the group, *AtSULTR3;5*, is plasma membrane localized and cooperates in roots with the low affinity transporter *AtSULTR2;1* under sulfur deficiency to increase sulfate uptake capacity for root-to-shoot vascular transport [55]. The Tal2g target *OsSULTR3;6* is most similar to *AtSULTR3;5* (57% identity) yet is expressed, in the absence of Xoc infection, primarily in seeds during later stages of seed development [42]. The physiological consequence of the recruitment of high *OsSULTR3;6* expression to mesophyll cells by Tal2g is therefore challenging to predict. Given the M27 phenotype, an attractive hypothesis is that it alters antioxidant capacity, impinging on redox signaling to dampen defense and allow more rapid induction of watersoaking by the pathogen. Another possibility is that it enhances watersoaking more directly, either through a redox-controlled mechanism or simply by altering osmotic equilibrium.

In *L. japonica*, the group 3 sulfate transporter gene *sst1*, which is more similar to *OsSULTR3;6* (56% identity) than to any other member of the gene family in rice, is essential for normal nodule development and symbiotic nitrogen fixation [56]. Its ortholog in poplar (*Populus trichocarpa*), PtSultr3;5, is among most highly induced transcripts during the establishment of symbiosis with the fungus *Laccaria bicolor* [57]. This gene is also strongly induced during both compatible and incompatible interactions with the poplar rust pathogen *Melampsora larici-populina* [58]. Whether the Tal2g target and these orthologs play analogous roles in such diverse plant-microbe interactions awaits in-depth functional analysis.

That a major *S* gene for leaf streak is a member of a large gene family recalls the situation in blight, in which five members of the large *OsSWEET* family can functionally substitute for one another as *S* genes, three so far have been shown to play that role, and distinct TAL effectors from multiple strains have been identified as the activators of two [11], [13], [19]. Whether any of the five other group 3 paralogs, or of the other 13 total members of the sulfate transporter gene family in rice can substitute for *OsSULTR3;6*, and whether any are actually targeted by other strains of Xoc is an important question. A tendency for *S* genes to be members of functionally analogous gene families would make sense from an evolutionary perspective, both for the advantage it would afford the pathogen by providing alternate targets should *cis*- (e.g. *xa13*) or *trans-* (e.g., *Bs3*) acting types of resistance be encountered, as well as the possibility it would afford the host to adapt through promoter mutation and resist targeting while maintaining essential functions. These processes might indeed drive one another [46]. On the other hand, if *OsSULTR3;6* is uniquely capable among its paralogs of serving as an *S* gene, the likelihood of identifying moderately stable resistance by screening for or engineering promoter variants that retain endogenous function is increased.

### General characteristics of TAL effector-driven gene expression

*OsSULTR3;6* was one of the most strongly induced and highly expressed genes in Xoc inoculated plants, as *OsSWEET11* was in Xoo inoculated plants. These major S genes were exceptional, with the majority of TAL effector targets being induced moderately. The blight *S* genes *OsTFIIAγ1* and *OsTFX1*, which contribute only moderately, were induced relatively weakly. Whether these differences reflect an evolutionary optimization of transactivation for major *S* genes, or gene specific differences in induction potential or optima, or chance, is unclear. The general pattern of relatively high basal expression and moderate fold increase across all identified TAL effector targets may be dominated by so-called collateral targeting inconsequential to disease and under no selection, and it suggests that TAL effectors may act as transcriptional enhancers more readily than as activators. However, the low variation in normalized expression levels for all targets at 96 h after inoculation suggests that on average, this enhancement is close to saturating.

We generally did not observe significant expression changes at early time points (*i.e.* 2 h and 4 h), possibly as a result of a low signal∶noise ratio caused by variation among the replicates, but expression of TAL effector targets generally increased steadily across the later time points. Though some genes were expressed at lower levels in Xoc- or Xoo-inoculated plants than in mock-inoculated plants at 96 h, no significant patterns of downregulation across all time points were observed. We tentatively conclude from these observations that TAL effectors of Xoc and Xoo do not significantly downregulate any genes in their host, despite the potential to do so through indirect effects, or theoretically, through nonfunctional binding that interferes with endogenous expression.

### False positives in target prediction

The average number of candidate EBEs in the rice promoterome, per TAL effector across all Xoc and Xoo TAL effectors, was 671. After excluding candidate EBEs in genes not upregulated after inoculation, that average dropped to 1.5, with some TAL effectors having none and some as many as seven. Nearly half of the filtered EBEs that were tested further (i.e., those for Xoc TAL effectors) were real. Thus, combining candidate EBE search results with global gene expression data is a robust and effective approach to identifying TAL effector targets.

Nevertheless, the method still yielded roughly as many false positives as true targets. Though upregulated during infection, false positives might include genes with EBEs that match but are inaccessible or in the wrong context to be functional, or genes with EBEs that score below the cutoff but are not actually sufficiently high affinity sites. In an attempt to decrease the number of falsely predicted targets and improve the efficiency of target identification in the future, we applied machine learning to our set of 24 real (Xoc and Xoo) and 20 falsely predicted (Xoc) targets (Table S7) using several characteristics of their candidate EBEs. The best classifier that resulted calls 22 of the real targets and three of the falsely predicted targets as real, for a recall of 0.92 and a precision of 0.88. Thus, it eliminates 85% (17/20) of the falsely predicted targets at a cost of less than 10% (2/24) of the real ones. The training set was relatively small, so these metrics may not hold strictly when applied to larger numbers of predicted targets, and even if they are relatively stable, if the goal is to comprehensively capture real targets, the classifier clearly can not be used as a strict filter. It is also important to remember that the classifier was trained only on EBEs that passed the score cutoff and were located in up-regulated genes, so performance metrics might not hold if the classifier is used on EBEs that do not meet these requirements. Rather, the probability this classifier provides should be used to prioritize already predicted targets for experimental validation (see Supporting Information for the Weka model file for the Naive Bayes classifier trained on all EBE features). Training on a greater number of targets as they are identified will improve both precision and recall, possibly even uncovering conditional relationships among characteristics of EBEs in real targets that the classifier currently calls incorrectly. With more targets, further comparison of classifiers built on subsets of EBE features might also reveal a smaller set of the most biologically relevant features that are sufficient to effectively discriminate real targets. Even with the small training set used here, the only slightly lower recall of the classifier based just on distance to transcriptional start site strongly suggests a major role for this feature.

As demonstrated by the results of our functional characterization of Tal2g EBEs and candidate EBEs (Figure 7), an important remaining challenge to eliminating false positives is a more nuanced understanding of TAL effector DNA binding. In particular, being able eventually to replace the RVD-nucleotide association frequency-based scoring matrix with one based on biochemically defined contributions of different RVD-nucleotide pairings, weighted to account for effects of position 5′ to 3′, will improve initial candidate EBE calling. Defining specificities for as many rare RVDs as possible will also be important to eliminate false positives and capture real targets for proteins like Tal2g. In this regard, we improved our predictions by substituting values of common RVDs for two rare ones, based on inference from structural data, and supported in the case of ‘SN’ by an experimental study [59].

### False negatives in target prediction

Better understanding of TAL effector DNA interactions will also help eliminate false negatives. Without the scoring substitutions for the rare RVDs in Tal2g, one of its targets, the monocopper oxidase gene, would have been overlooked. Another example is suggested by the lack of identified targets for either Tal11a or Tal11b despite the reduced virulence of *tal11* mutants (recalling however that complementation analysis was not performed to verify a role for either effector). A very low level of induction may be sufficient for function of some targets, such as an *R* gene like *Xa27* [22] or an *S* gene that encodes a transcription factor, so false negatives could derive from a failure to detect differential expression in the initial transcript profiling experiment. A false negative could also result for a TAL effector with lax specificity. *Xa27* again serves as an example. AvrXa27 contains at several positions an RVD with dual or no specificity; its EBE in *Xa27* ranks 5,368^th^ in the rice promoterome, nestled above the low-scoring outlier cutoff [26]. Exclusion of sites preceded by any base other than T, as specified in our search, might also pass over a real EBE. The TalC EBE in *OsSWEET14*, discussed in the introduction, is a salient if rare example. Two additional, theoretical examples are worth considering. The first is a gene whose expression is activated via read-through transcription by a TAL effector that targets a neighboring gene upstream. The second is a gene for which overall transcript levels do not change detectably, but which yields a unique alternative transcript when driven by the TAL effector due to TAL effector-dependent repositioning of the transcriptional start site [41], [60]. Transcript profiling by next generation sequencing of cDNA (RNAseq) [e.g., 61], in contrast to the GeneChip expression experiment that began this study, should provide the sensitivity to detect weakly expressed or weakly induced genes as well as alternative transcripts, to reduce or eliminate false negatives that might otherwise result. Regarding TAL effectors with lax specificity, EBEs with a non-canonical preceding base, and potential read-through targeting, adjusting EBE search parameters is a simple solution, but will unavoidably increase the number of false positives.

### Direct vs. indirect targets

Given the current understanding of TAL effector function and the ability to predict binding sites using the code, we considered each gene that was activated by a TAL effector and that displayed a strong candidate EBE for that effector to be directly activated. Yet even meeting these criteria, it is formally possible that such a gene might be activated indirectly, i.e., in response to expression of another gene directly activated by the TAL effector. In pepper, prior to discovery of the code, direct targets of AvrBs3 were isolated by screening for transcripts whose upregulation by AvrBs3 occurs even in the presence of the eukaryotic translation inhibitor cycloheximide [14], [16]. To address the possibility that some of the Xoc TAL effector targets we identified in rice are indirect targets, using RT-PCR we tested Xoc-triggered transcript accumulation of the targets for sensitivity to cycloheximide, measured at 8, 16, 24, and 36 hr after co-infiltration (Figure S5). At the two earlier time points transcripts of all but one target accumulated similarly in response to Xoc with or without cycloheximide, and most showed identical patterns across all four time points. However, several showed distinct patterns of up and down regulation across the time points in response to cycloheximide treatment alone. Furthermore, cycloheximide treatment strongly and persistently upregulated three pathogenesis-related genes previously observed to be induced by biotic stresses, included as controls, and transiently induced a fourth. Induction of an additional control, *Os05g42150*, which is the most significantly Xoc-induced gene in our dataset (Table S1) and is not predicted to be a TAL effector target, was unaffected by cycloheximide at the two earlier time points and was slightly repressed at the later ones. The results overall thus reveal differing and confounding epistatic effects of cycloheximide treatment in rice that render conclusive identification of direct and indirect targets by this method impossible.

Regarding the single target showing repression of Xoc-induced transcript accumulation in the presence of cycloheximide, the monocopper oxidase gene *Os06g46500*, in addition to its being upregulated during infection and harboring an appropriately positioned strong candidate EBE for Tal2g, several other lines of evidence support it being a direct target. First, in the context of the Bs3 promoter, tested in *N. benthamiana*, that EBE is functional (Figure 7). Second, the pattern of induction of *Os06g46500* by Xoc is rapid and robust, virtually identical to that of the SO_4_ transporter gene targeted by Tal2g (Figure S6) and similar to the patterns displayed by the verified Xoo TAL effector targets (Figure 2). Third, no other Tal2g target that might activate *Os06g46500* was predicted other than the SO_4_ transporter gene, and activation of the SO_4_ transporter gene by dTALEs was not accompanied by activation of *Os06g46500* (Figure 6B). Also, activation of *Os06g46500* itself with a dTALE targeting a site in the vicinity of the native EBE (Figure 6) indicates that the promoter is not generally inaccessible to binding by a TAL effector. Finally, assuming that if not all, at least most of the targets in the training set for the classifier we generated by machine learning are direct targets, the leave-one-out validation tests showed that *Os06g46500* shares the characteristics of those targets (including the previously confirmed targets of Xoo TAL effectors).

For *Os06g46500*, and the rest of the targets we identified here, assaying activation following disruption of the endogenous EBEs by site-directed mutagenesis would provide the most direct evidence for or against direct targeting, but such experiments are beyond the scope of the present study. Absent such data, it remains possible that some of the targets we identified are indirect ones. For the reasons detailed in the above two paragraphs, we conclude that this is unlikely, but to the extent that it were true, it would affect the utility of our predictive classifier.

### Outlook

Many crops, including rice, wheat, cotton, citrus, tomato, cassava, soybean, and others, suffer losses due to *Xanthomonas spp.* that deploy TAL effectors. We demonstrated here that TAL effector activity in bacterial leaf streak of rice is directly responsible for nearly a quarter of the gene activation detected during infection. Considering the likely downstream effects, the total proportion is certain to be even greater. Our study provides new insight into bacterial leaf streak of rice in relation to bacterial blight and identifies a major new S gene, but TAL effector target identification in several pathosystems is a critically important ongoing objective. Probing the diversity and functions of TAL effector activated *S* and *R* genes will expand our knowledge of disease and defense mechanisms, and our ability to exploit those mechanisms for effective disease control. Patterns of distribution of different *S* genes in diverse pathosystems might yet reveal causal distinctions between pathogens that colonize the mesophyll and those that invade the xylem. New targets will also refine our understanding of functional TAL effector-DNA interactions, improving our ability to use these proteins [62]. Though improvements can be made, and challenges remain, the overall combined transcriptomic and computational approach we successfully undertook constitutes a moderately high throughput method that can be applied to TAL effector target identification in many *Xanthomonas*-host interactions, particularly as more, complete pathogen and host genome sequences become available.

## Materials and Methods

### Plant material and growth conditions

*Oryza sativa* ssp. *japonica* cv. Nipponbare plants were grown in LC-1 soil mixture (Sungro, Bellevue, WA) in PGC15 growth chambers (Percival) in trays approximately 60 cm below a combination of fluorescent and incandescent bulbs providing approximately 1,000 µmoles/m^2^/s measured at 15 cm, under a cycle of 12 h light at 28°C and 12 h dark at 25°C. Fertilizer (Peters Professional, St. Louis, MO) and iron chelate micronutrient (Becker Underwood, Ames, IA) were applied with watering every two days at 0.25 and 4.5 g/l, respectively, until the day before inoculation. *Nicotiana benthamiana* plants were grown in LC-1 in a PGC15 growth chamber at approximately 90 cm below the lights, under a cycle of 16 h light (fluorescent lighting at 22°C, and 8 h dark at 18°C, and fertilized using a surface amendment of Osmocote granules (ScottsMiracle-Gro, Maryville, OH).

### Bacterial strains and plasmids used, culture, and transformation

Bacterial strains and plasmids used for this study are listed in Table S8. *E. coli* strains were grown in LB medium at 37°C and *A. tumefaciens* in YEP medium (10 g/l peptone, 10 g/l yeast extract, 5 g/l NaCl, 1.5% agar) at 28°C, and transformed by standard electroporation. *X. oryzae* strains were cultured in GYE (20 g/l glucose, 10 g/l yeast extract) at 28°C unless otherwise specified, and were transformed by electroporation as described previously [63], except that 1 µl (5 µg) TypeOne Restriction Inhibitor (Epicentre Biotechnologies, Madison, WI) was added prior. Antibiotics were used for selection as follows: ampicillin at 100 µg/ml, gentamycin at 25 µg/ml, kanamycin at 25 µg/ml, spectinomycin at 25 µg/ml, and tetracycline at 10 µg/ml for *E. coli* or 2 µg/ml for *X. oryzae*.

### GeneChip expression experiment

#### Experimental design

The experiment was carried out in four independent replicates, each one week apart. Plants were grown three trays together per replicate, one each for Xoc-, Xoo-, and mock-inoculation. Trays were moved to a new chamber once per week, in order through three chambers total, so that plants for each replicate were incubated in the same chamber (the third one) following inoculation. For the different replicates, position of the trays left to right within the chambers was maintained according to a random assignment specific to each replicate that was also used for the order of inoculation and tissue collection. Tissue was collected at 2, 4, 8, 24 and 96 hours after inoculation, with plants in each tray randomly assigned to time points. Overall, the experiment followed a split-plot design with inoculation as the whole-plot factor and time as the split-plot factor.

#### Inoculation

Plants were grown four per 5 cm square pot, arranged in trays of 20 pots, one tray for each inoculum. To allow inversion for inoculation (see below), each seed was sown through a short plastic tube (a five ml pipette tip with its tapered end removed) extending from the soil surface through a fiberglass lid (cafeteria tray) that had been perforated with appropriately spaced 1 cm holes and affixed to the top of the tray. Fourteen days after sowing, and 2 h after the beginning of the light period, trays (with seedlings projecting from the tubes) were inverted over a plastic dishpan containing 7 l of inoculum to submerge the seedlings for vacuum infiltration. This was carried out in a custom vacuum chamber by subjecting the submerged plants to 500 mm Hg for two minutes followed by a rapid return to atmospheric pressure, two consecutive times. Xoc and Xoo inoculum was prepared as follows. For each, a single colony from a fresh plate was transferred to 5 ml of liquid medium and incubated for 24 h at 28°C with constant shaking at 250 r.p.m. Then, 2 ml of this culture were transferred to 300 ml of fresh liquid medium and incubated as above for an additional 18 h. Cells were pelleted by centrifugation at 4,000× g for 10 min, washed twice and resuspended in sterile 10 mm MgCl_2_ to OD_600_ = 0.05. Tween-20 was added to a final concentration of 0.5%. Seven l of this suspension were used for inoculation. Mock inoculum consisted of 10 mm MgCl_2_ and Tween-20 only. Following inoculation, plants were returned to a growth chamber.

#### Tissue collection

Leaves were cut and pooled from plants in four pots (16 plants, approximately 2 g fresh weight) for each timepoint per inoculum per replicate. Harvested tissue was immediately frozen in liquid nitrogen and stored at −80°C until processing.

#### RNA isolation, probe synthesis, and hybridization

Total RNA was isolated using a hot (60°C) phenol/guanidine thiocyanate method [64]. Probe synthesis and labeling were performed at the Iowa State University GeneChip Core facility (Ames, IA, U.S.A.), using the One Cycle and GeneChip IVT labeling kits. Fifteen µg of fragmented cRNA was used to make each hybridization cocktail containing 10% dimethyl sulfoxide, and 10 µg equivalent was hybridized to the GeneChip Rice Genome Array (Affymetrix, Santa Clara, CA).

#### Data acquisition and analysis

Stained chips were immediately scanned with the GeneChip Scanner 3000 7G (Affymetrix). Scans were examined for any visible defects and satisfactory image files were analyzed to generate raw data files using the GeneChip Operating Software (GCOS v1.4.0.036; Affymetrix) with default settings. Robust multi-array analysis (RMA) [65], [66] was used to normalize the data. Note that RMA normalization includes a log_2_ transformation, so fold-change across absolute values for two normalized values X and Y is calculated as 2^(X-Y)^. A mixed linear model was fit separately to RMA-normalized data for each probeset. Each mixed linear model included fixed effects for replicate, treatment, time, and treatment-by-time interaction, as well as random effects for the trays. We used the model for each probeset to test for a difference between its patterns of expression over time in Xoc- and mock-, Xoo- and mock-, and Xoc- and Xoo-inoculated plants. The null hypothesis for each comparison was that the expression difference between inoculations was constant across all five time points. The 55,515 *p*-values from each of these three inoculation comparisons, representing all probesets, were converted to *q*-values using the method of Storey and Tibshirani [67]. To better enhance capture of genes that were moderately differentially expressed in this screening experiment, we used a relatively lax *q* value cutoff of 0.30. This implies that approximately 70% of the genes declared to be differentially expressed are expected to be true positives.

### GeneChip expression data access

GeneChip data are available at the PLEXdb gene expression resource (www.plexdb.org) [68] under accession OS3 and at NCBI-GEO under accession GSE16793.

### Prediction of TAL effector targets

Promoter sequences, defined as the 1,000 bases upstream of the start codon, for the approximately 56,000 rice genes annotated in the MSU Rice Genome Annotation Project Release 7 (http://rice.plantbiology.msu.edu/) were searched using our previously published TAL effector-target scoring model [32], for the best-scoring site in each promoter for each of the 40 unique *Xoo* and *Xoc* TAL effectors. Scoring takes the sum of the negative log probabilities of the RVD-nucleotide pairings at a site, so a lower score is a better score. Sites were required to be directly preceded by a 5′ T. The scoring matrix was used as published and separately with the RVDs ‘SN’ and ‘YG’ assigned nucleotide association frequencies of ‘NN’ and ‘NG’, respectively (see [Results]). The distributions of the approximately 56,000 resulting scores for each TAL effector in each case were then used to calculate a cutoff for outliers, defined as the 25^th^ percentile minus 1.5 times the interquartile range. Promoters were then rescanned to identify all sites scoring below (better than) that cutoff for each TAL effector, and those sites were retained as candidate EBEs. Finally, the list of candidate EBEs for each TAL effector was cross-referenced to the GeneChip expression data. Candidate EBEs in promoters of genes upregulated in Xoc- or Xoo-inoculated plants relative to mock were taken as predicted targets.

### Generation of *X. oryzae* pv. oryzicola BLS256 TAL effector gene knockout mutants

A library of *tal* gene knockout strains of Xoc was generated by transformation with the suicide (non-replicative) plasmid pSM7 (Figure 4A; Table S8) [69], pSM7 harbors a 4.5-kb *Pst*I fragment containing all but the first 80 bp of the ORF of *tal* gene *aB4.5* [12] with an insertion of the EZ-Tn5 <*Not*I/KAN-3> transposon (Epicentre) at bp 1,769 of the gene, in repeat number 9 of 17.5 (sequence available on request). The vector is pBluescript II KS(+) (Agilent Technologies, Santa Clara, CA). The transposon provides kanamycin resistance. Selection for this marker yields strains in which the cloned, mutated *tal* gene has undergone homologous recombination with an endogenous *tal* gene. Because the *tal* ORF is truncated at the 5′ end, either a single or double recombination that retains the transposon results in a *tal* gene knockout. Double recombination can knock out clustered *tal* genes. The 4.5 kb *Pst*I fragment also includes the first 85 bp of the *avrXa10 tal* gene downstream of *ab4.5*, which might increase the likelihood of complex recombination. To determine the number of insertions per strain and to map insertions, genomic DNA was extracted using the GenElute Bacterial Genomic Kit (Sigma-Aldrich, St. Louis, MO). Strains with single insertions were identified by Southern blot using EZ-Tn5 <*Not*I/KAN-3> as a probe. Insertion endpoints were mapped by amplifying and sequencing the distal ends of 5′ and 3′ fragments flanking the transposon and extending outside the repeat region. Primers used for amplifying 5′ flanking DNA were forward primer p369 (5′-TTCTGfCCCGGACCCCAACCGGATAG), matching a conserved 5′ sequence in Xoc *tal* genes, and reverse primer p395 (5′-TCCCGTTGAATATGGCTCATAACACCCC), corresponding to the transposon. For the 3′ fragment, forward primer p397 (5′-GTCCACCTACAACAAAGCTCTCATCAACC), corresponding to the transposon, and reverse primer p398 (5′-TCCTCTTCGTTGAATGCC), matching a conserved 3′ sequence of Xoc *tal* genes, were used. Sequencing of the distal ends of the 5′ and 3′ amplicons (furthest from the repeat region) was done using *tal* gene plus-strand primer p396 (5′-ACCCCAACCGGATAGG) and p398, respectively. In all but a few cases, insertion endpoints were unambiguously identified by polymorphisms among the 5′ and 3′ sequences of the 26 Xoc *tal* genes and two *tal* pseudogenes (Figure 4).

### Cloning of TAL effector genes of *X. oryzae* pv. oryzicola BLS256

Two micrograms of genomic DNA of *X. oryzae* pv. oryzicola strain BLS256 were digested with *Bam*HI and separated in 1% agarose by electrophoresis. DNA fragments from 2 to 5 kb were gel purified and ligated into pBluescript II SK- (Agilent) previously digested with *Bam*HI and dephosphorylated with alkaline phosphatase (CIP; New England Biolabs, Ipswitch, MA). The ligation reaction was then used to transform *E. coli* TOP10 cells, and colonies harboring TAL effector clones were identified by colony PCR using oligonucleotides p270 (GCCAAGTCCTGCCCGCG) and p271 (CCTCCAGGGCGCGTGC), which target the conserved 5′ region of *Xanthomonas oryzae* TAL effector genes. The *tal* gene fragments in these clones were tentatively identified based on size and 5′ and 3′ sequencing. Next, the corresponding *Sph*I fragment of each *tal* gene, encoding the repeat region and short flanking sequences, was cloned into the *tal1c* backbone (*i.e.*, lacking the corresponding *Sph*I fragment) in the entry vector pCS466 [63] and confirmed by 5′- and 3′- sequencing with oligos p235 (GGAGGCCTTGCTCACGGATGC) and p236 (GGCCGGTGACAGCACGATCCG), respectively. For *tal2g*, *tal4a*, and *tal8*, *Bam*HI fragments were cloned into pCS466 (cut with *Bam*HI) instead, since those genes are each missing one of the *Sph*I sites. The reconstituted genes in pCS466 were then recombined into the broad host-range destination vector pKEB31 [27], using Gateway LR Clonase (Life Technologies, Carlsbad, CA), for expression in *Xanthomonas*.

### RT-PCR

Xoc strains were cultured for 3–4 days on solid media then resuspended in 10 mM MgCl_2_ to OD_600_ = 0.5 (approximately 1×10^8^ CFU/ml) and infiltrated into the abaxial surface of fully expanded leaves of 4-week old rice plants using a needleless syringe. 10 mM MgCl_2_ alone was infiltrated as the mock. Infiltrated tissue was collected at 48 h and RNA prepared using the RNeasy Mini Kit (Qiagen, Valencia, CA). Before elution, RNA was subjected to in-column digestion with the RNase-Free DNase Set (Qiagen). Two µg of total RNA were used for first-strand cDNA synthesis using SuperScript III reverse transcriptase (Life Technologies) and standard oligo dT_20_. Reverse transcriptase reactions were diluted 5 times and 1 µl was used as a template for PCR with Phire Hot Start II DNA polymerase (Thermo Scientific, Waltham, MA) together with transcript-specific oligonucleotides for 30 sec at 98°C, followed by 23–25 cycles (depending on transcript abundance) of 10 sec at 98°C, 5 sec at 60°C, and 10 sec at 72°C. The oligonucleotides used are listed in Table S9.

### Virulence assays and quantification of bacterial populations

Rice leaves were inoculated by syringe infiltration as described above for RT-PCR. Virulence was quantified at the specified days after inoculation as lesion expansion, in mm, from the inoculation spot (Figure 4B). To measure bacterial populations, duplicate sets of three leaves per treatment per time-point were collected. One set was used to quantify total bacterial populations and the other to quantify surface bacteria. For total bacterial counts, 10 cm leaf sections centered on the infiltration spot or leaf sections as indicated in Figure 6D were cut into small pieces and ground thoroughly in 2 ml of water using a mortar and pestle. For surface bacteria, a leaf section encompassing the watersoaked area was washed with 50 µl of water twice and the wash diluted into 1 ml of water. Samples were thereafter diluted serially in sterile water and spotted on peptone sucrose agar (10 g/l sucrose, 10 g/l peptone, 1 g/l sodium glutamate, 1.5% agar) supplemented with cephalexin at 20 µg/ml. Plates were incubated at 28°C until appearance of single colonies, and colonies at the dilution they were first distinct were counted. For each replicate sample, eight such measurements were made. Results are displayed as the mean and standard deviation of all measurements for all replicates. Experiments were repeated at least three times with consistent results.

### Designer TAL effectors

TAL Effector Targeter [32] was used to target designer TAL effectors (dTALEs) to the promoter regions of *Os01g52130* and *Os06g46500*. dTALEs were assembled by golden gate cloning into the entry vector pTAL1 as described [27] and subsequently transferred to the broad host range destination vector pKEB31 [27] by Gateway LR Clonase (Life Technologies). RVD sequences if the dTALEs used in this are provided in Text S1.

### GUS reporter gene assay of TAL effector activity

GUS reporter assays were conducted in *Nicotiana benthamiana* leaves of five-week old plants (from the date of sowing) using the substrate 5-bromo-4-chloro-3-indoyl glucuronide (X-Gluc) as described [70], using three leaf discs from different plants per treatment, collected at 48 hours after infiltration of *Agrobacterium*. Experiments were repeated twice. Determination of total protein in sample extracts was performed using the Bradford assay kit (Bio-Rad). The vector for T-DNA delivery of *avrBs3* under the 35S promoter was pGWB5-*avrBs3* [40]. The equivalent construct for *tal2g*, pGWB5-*tal2g*, was made by replacing the ∼3.3 kb *Bam*HI fragment of an *avrBs3* clone in the entry vector pENTR-D (Life Technologies; gift of T. Lahaye, University of Munich) with the ∼3.2 kb *Bam*HI fragment of *tal2g*, then moving the reconstituted *tal2g* equivalent gene to the binary destination vector pGWB5 [71] using Gateway LR Clonase (Life Technologies). The pGWB5 derivatives were introduced into *Agrobacterium tumefaciens* strain GV3101 by electroporation; transformants were selected with 25 µg/ml each of kanamycin and gentamycin. The reporter constructs were made by first PCR amplifying from a longer *Bs3* promoter clone (gift of T. Lahaye) the AvrBs3-responsive 343 bp sequence upstream of the *Bs3* start codon, using previously reported primers [70] and inserting it into the Gateway entry vector pCR8/TOPO-TA (Life Technologies). A single base substitution was then introduced by site directed mutagenesis (Agilent) to create an *Nco*I site 47 bp upstream of the native EBE for AvrBs3. Candidate Tal2g EBEs flanked upstream by 5 bp and downstream by 4 bp matching their native context were synthesized as double stranded oligonucleotides with *Nco*I overhangs (Text S1) and cloned into the *Nco*I site of the modified *Bs3* promoter. Finally, the modified *Bs3* promoter and derivatives were transferred into the binary GUS reporter vector pGWB3 [71] using Gateway LR Clonase (Life Technologies), and the resulting plasmids introduced into *A. tumefaciens* GV3101 as described above.

### Construction and validation of machine learning classifiers

Both Naive Bayes and logistic regression classifiers were implemented using Weka 3.6.9 [72] with default options, which select the discrimination threshold that maximizes F measure. All classifiers were trained on the candidate EBEs in Table S7 that were determined to be either in real or falsely predicted targets (“Yes” or “No” in column P, “Verified”). Classifiers were trained using various subsets of the following features: relative score, actual score, rank, distance to TXS, distance to TLS, proximity to a TATA box, and distance to a Y Patch. If a predicted EBE was located in a promoter without a TATA box or without a Y patch, or with no annotated TXS, the value for that feature was considered missing and replaced with a question mark. All classifiers were evaluated using leave-one-out cross validation. The receiver operating characteristic curve and precision recall curve in Figure S3 were plotted using the ROCR package [73].

## Supporting Information

Figure S1 **Experimentally verified targets of** ***X. oryzae*** **pv. oryzicola BLS256 (Xoc) TAL effectors in rice: results of RT-PCR analyses to test specific dependence of induction on the TAL effector.** Targets (and actin, which was used as an internal control to normalize cDNA amounts) are indicated at far right; “Os_LOC” is omitted from locus IDs. *Xanthomonas axonopodis* pv. glycines strain EB08 (Xag) was used to deliver individual Xoc TAL effectors and test their sufficiency for induction of respective predicted targets, and Xoc *tal* gene knock-out mutants were used to test the requirement of each TAL effector for target induction. Xag transformed with, from left to right, vector pAC99 carrying the gene for the TAL effector being tested, another *tal* gene as a specificity control, or no *tal* gene (–) were used. For Xoc, from left to right, the wildtype (WT), a marker exchange mutant with a disruption of the gene for the test TAL effector and transformed with the empty vector pAC99, that mutant transformed with pAC99 carrying the intact *tal* gene (designated in parentheses) cloned in pAC99 for complementation, a type III secretion-deficient Xoc derivative (*hrcC^−^*), and an independent *tal* gene mutant as a specificity control were used, except that no Xoc inoculations were done for Tal2a targets because no *tal2a* mutant was obtained. Plant tissue for RNA preparation was harvested at 48 h after infiltration and actin was used as internal control to normalize cDNA amounts. Experiments were repeated multiple times including samples collected at 72 h after infiltration and showed identical results.
(PDF)

Figure S2 **Functionality of Tal2c and Tal2d in the M27 mutant derivative of** ***X. oryzae*** **pv. oryzicola BLS256.** Shown is accumulation of transcripts of the Tal2c and Tal2d targets (Table 1 and Table S7), and the two Tal2g targets for reference, in rice at 48 hr after infiltration with wild type (WT), M27, or the type III secretion-deficient *hrcC^−^* mutant strain, determined by RT-PCR. Actin transcript was included as a control to normalize cDNA amounts. Experiments were repeated twice showing consistent results.
(PDF)

Figure S3 **Performance of a Naive Bayes classifiers trained on all EBE features or a logistic regression classifier trained on distance to transcriptional start site (TXS) using leave-one-out cross validation.** (A) Receiver operating characteristic curve. (B) Precision and recall.
(PDF)

Figure S4 **Lower hydrogen peroxide levels in rice leaves infiltrated with** ***X. oryzae*** **pv. oryzicola BLS256 (Xoc) compared to** ***X. oryzae*** **pv oryzae PXO99^A^ (Xoo)- or mock-treated leaves.** Hydrogen peroxide activity was determined in 10 cm leaf segments 4 days after infiltration with Xoc, Xoo, or water (Mock), using a chemiluminescence method [1]. The difference between catalase-treated and non-treated samples was considered a relative measure of H_2_O_2_. Values are averages of three replicates. Vertical bars show standard deviation.
(PDF)

Figure S5 **Effects of the protein synthesis inhibitor cycloheximide (CHX) on expression kinetics of targets of** ***X. oryzae*** **pv. oryzicola BLS256 (Xoc) TAL effectors.** Shown are results of RT-PCR performed on rice (cv. Nipponbare) leaf tissue harvested at 0, 8, 16, 24, and 36 hours after infiltration (hai) with Xoc, Xoc plus 50 µM CHX, or 50 µM CHX alone. Targets are indicated at right, by locus ID, omitting the prefix “LOC_Os”. Pathogenesis-related genes *PR1a* (*07g03710*), *PR1b* (*01g28450*), *PAL* (*02g41630*), and *EL2* (*03g01740*), previously observed to be induced by biotic stresses [2]–[4] and *05g42150*, the most significantly Xoc-induced gene in our dataset (Table S1) and not predicted to be a TAL effector target, were used as controls for the effect of CHX treatment. An actin gene, which was insensitive to any treatment, is included as a reference for relative transcript abundance across samples. Experiments were repeated once with 50 µM and once with 100 µM CHX using the 24 time point, and showed similar results.
(PDF)

Figure S6 **Expression patterns of the two targets of Tal2g,** ***Os06g46500*** **and** ***Os01g52130*** **, in the GeneChip experiment.** Results are plotted as in Figure 2.
(PDF)

Software S1 Weka (3.6.9) model file for Naive Bayes classifier trained on all EBE features.
(MODEL)

Table S1 **Rice (cv. Nipponbare) genes differentially expressed over time (q-Value ≤0.3) in response to inoculation with** ***Xanthomonas oryzae*** **pv. oryzicola BLS256.**
(XLSX)

Table S2 **Rice (cv. Nipponbare) genes differentially expressed over time (q-Value ≤0.3) in response to inoculation with** ***Xanthomonas oryzae*** **pv. oryzae PXO99^A^.**
(XLSX)

Table S3 **Rice (cv. Nipponbare) genes differentially expressed over time (q-Value ≤0.3) in response both to inoculation with** ***Xanthomonas oryzae*** **pv. oryzicola BLS256 Xoc) and** ***X. oryzae*** **pv. oryzae PXO99^A^ (Xoo).**
(XLSX)

Table S4 **Ontology of rice (cv. Nipponbare) genes induced by** ***Xanthomonas oryzae*** **pv. oryzicola BLS256.**
(XLSX)

Table S5 **Ontology of rice (cv. Nipponbare) genes induced by** ***Xanthomonas oryzae*** **pv. oryzae PXO99^A^.**
(XLSX)

Table S6 **Rice (cv. Nipponbare) genes induced by** ***Xanthomonas oryzae*** **pv. oryzicola BLS256 related to detoxification of reactive oxygen species and to redox status control.**
(XLSX)

Table S7 **All computationally predicted targets in rice (cv. Nipponbare) of TAL effectors of** ***Xanthomonas oryzae*** **pv. oryzicola BLS256 (Xoc) and TAL effectors of** ***Xanthomonas oryzae*** **pv. oryzae PXO99^A^ (Xoo).**
(XLSX)

Table S8 **Bacterial strains and plasmids used.**
(XLSX)

Table S9 **Primers used for RT-PCR amplification of selected rice gene transcripts.**
(XLSX)

Text S1 **Supporting information for Materials and Methods.**
(PDF)
