# Correction: Code-Assisted Discovery of TAL Effector Targets in Bacterial Leaf Streak of Rice Reveals Contrast with Bacterial Blight and a Novel Susceptibility Gene

**DOI:** 10.1371/journal.ppat.1004126

**Published:** 2014-04-16

**Authors:** The PLOS Pathogens Staff

There are data and labeling errors in Figure 3. In the original Figure 3, the gel image for *Os07g06970* is incorrect. It is a duplicate of the image for *Os01g40290*. The new Figure 3 contains the correct image. Also, the original Figure 3 was generated using Locus IDs from Release 5.0 of the Rice Genome Annotation Project. Relative to Release 7.0, used for all other reporting in the article, one of these Locus IDs, *Os10g38495*, is obsolete. It has been updated in the new Figure 3 to the corresponding Release 7.0 Locus ID, *Os10g38489*. The remaining Locus IDs are the same in Release 5.0 and Release 7.0 and unchanged in the new Figure 3.

**Figure 3:**
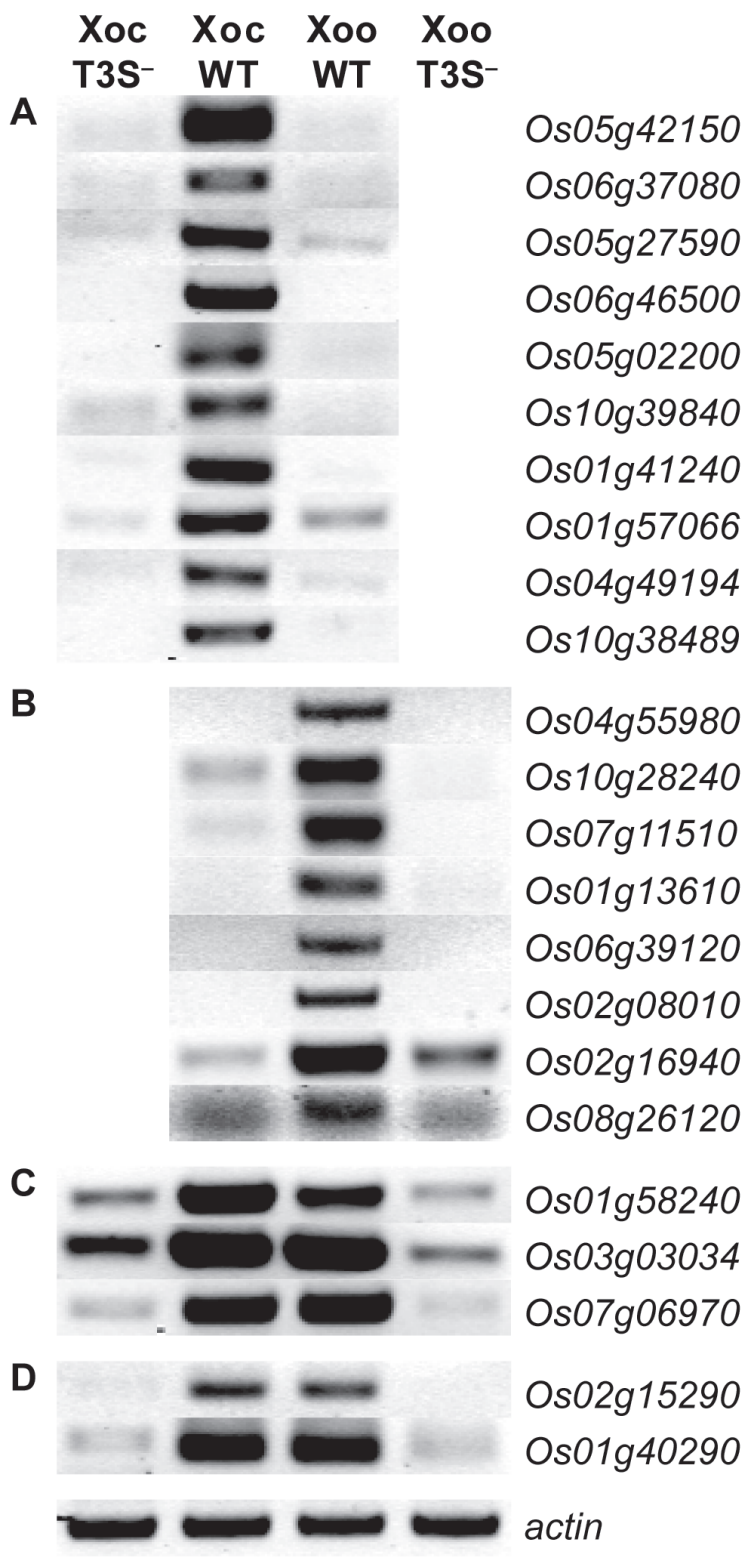
Type III secretion system dependence of the most significant rice gene expression changes. RT-PCR results reflecting transcript abundance are shown for rice genes identified by GeneChip expression analysis as the ten (or fewer) most significantly differentially expressed in response to (A) *X. oryzae* pv. oryzicola BLS256 (Xoc), (B) *X. oryzae* pv. oryzae strain PXO99^A^ (Xoo), (C) Xoc and Xoo similarly, or (D) Xoc and Xoo to different extents. Leaf samples were harvested at 36 hours after inoculation with wild-type strains or with the type III secretion (T3S^−^) deficient derivatives. RT-PCR results for previously reported Xoo-induced genes, *OsSWEET11* and *TFIIAγ1* [9], [10], are omitted. An actin gene (*Os04g57210*) that is not differentially expressed was used as a reference for relative transcript abundance across samples. The experiment was repeated twice and yielded the same results.

Please see the corrected Figure 3 here. The legend remains unchanged.

There is an error in Figure 2, Table S1, Table S6, and Table S9. Release 7.0 Locus ID *Os10g38489* correctly corresponds to *Os10g38495* from Release 5.0, *i.e.* to probeset Os.2612.1.S1_at. However, the Release 7.0 Locus ID recorded for data associated with this probeset in Figure 2, Table S1, Table S6, and Table S9 is the paralog *Os10g38640* and is incorrect. Os.2612.1.S1_at does not represent *Os10g38640*. *Os10g38640* should be replaced with *Os10g38489* in Figure 2, Table S1, Table S6, and Table S9, in each of which it occurs exactly once. The primers listed for *Os10g38640* in Table S9 correspond uniquely to *Os10g38489*. They were designed for the Release 5.0 gene model at that coordinate, *Os10g38495*, and should be left unchanged.

There is an additional error in Figure 2 and Table S1, that is also in Table S4. In Figure 2, Table S1, and Table S4, the Release 7.0 Locus ID recorded for data associated with probeset Os.46631.1.S1_x_at is *Os07g29750* and is incorrect. Os.46631.1.S1_x_at does not represent *Os07g29750.* The correct Locus ID for Os.46631.1.S1_x_at is the paralog *Os10g39840*. *Os10g39840* is unchanged from Release 5.0 to Release 7.0 and appears correctly in Figure 3. *Os07g29750* should be replaced with *Os10g39840* in Figure 2, Table S1, and Table S4, in each of which it occurs exactly once.

There is additional information to add to Table S9. Primers used to amplify *Os10g39840* (to generate the corresponding data in Figure 3) are (5’ to 3’) forward primer CCGATCAGGAGGTACGAGAAGAAGG and reverse primer GCACGCCTCAACTACCAAATTGC.

There are two errors in Table 1 and Table S7. *Os10g38489*, unlike *Os10g38640*, is a predicted target, displaying a candidate EBE for Tal1b of Xoc. *Os10g38489* with associated data is added to the corrected Table 1 and corrected Table S7. *Os07g29750* is incorrectly included in Table 1 and Table S7 as a predicted target of Xoc Tal4c. Based on the probeset that correctly maps to *Os07g29750*, OsAffx16482.1.S1_x_at, *Os07g29750* is not differentially expressed in any pairwise comparison, and therefore would not be considered a predicted target, despite its candidate EBE for Tal4c. *Os07g29750* is removed from the new Table 1 and Table S7. It is not be replaced in these tables with *Os10g39840* because *Os10g39840* displays no candidate EBE for any Xoc TAL effector. There is therefore no net change in the total number of predicted targets.

**Table 1:** Predicted *X. oryzae* pv. oryzicola BL256 TAL effector targets in rice (cv. Nipponbare) induced during infection and results of verification experiments.^a^

TAL effector	Target Locus ID^b^	Probe set ID(s)	Fold change 2-96h Xoc^c^	Fold change Mock-Xoc 96h^d^	*q* (Mock- Xoc)^e^	EBE Score^f^	EBE rel. score^g^	EBE rank^h^	EBE to TLS^i^	EBE to TXS^j^	EBE to TATA box^k^	EBE to Y patch^l^	Induced by		Description
													*tal* gene knockout strain of Xoc^l^	Xag expressing the *tal* gene^m^	
Tal4a	01g27210	Os.7911.1.S1_at	1.63	1.66	1.8E-01	29.22	2.85	341	253	143	-50	none	+	-	Glutathione S-transferase, putative, expressed
Tal6	01g31220	Os.6438.1.S1_a_at Os.6438.2.S1_x_at	1.48 1.48	1.53 1.52	8.0E-02 1.1E-02	18.75	2.38	685	157	152	none	33	-	+	Expressed protein
Tal2d	01g51040	Os.53457.1.S1_at	2.30	2.23	1.9E-01	14.32	2.19	324	527	none	-328	0	+	-	Transmembrane protein 16K, putative, expressed
Tal9b	01g51040	Os.53457.1.S1_at	2.30	2.23	1.9E-01	14.07	2.81	275	18	0	-299	none	-	+	Transmembrane protein 16K, putative, expressed
Tal2g	01g52130	Os.41841.1.S1_at	13.00	9.59	1.3E-06	13.94	1.97	77	427	58	28	none	-	+	Sulfate transporter, putative, expressed
Tal3b	01g53220	Os.35681.1.S1_at	3.50	4.12	2.2E-06	17.72	2.92	611	146	-5	-137	none	nd	nd	HSF-type DNA-binding domain containing protein, expressed
Tal6	02g14770	Os.2450.1.S1_a_at Os.2450.3.S1_x_at	1.88 1.85	1.77 1.55	1.3E-02 6.8E-02	18.48	2.35	569	92	48	-70	-37	+	-	Phosphoenolpyruvate carboxylase, putative, expressed
Tal11a	02g15290	Os.56119.1.S1_at	1.72	4.93	4.1E-07	20.15	3.18	582	422	none	-288	none	+	-	VQ domain containing protein, putative, expressed
Tal5a	02g15290	Os.56119.1.S1_at	1.72	4.93	4.1E-07	23.32	1.88	107	148	30	-3	-180	-	+	VQ domain containing protein, putative, expressed
Tal7	02g15710	OsAffx.2629.1.S1_at	5.70	5.52	8.4E-02	15.75	1.94	265	951	none	150	434	nd	nd	Plastocyanin-like domain containing protein, putative, expressed
Tal3b	02g34970	Os.47735.1.S1_at	9.07	5.31	4.0E-07	15.33	2.53	75	110	29	-282	none	-	+	No apical meristem protein, putative, expressed
Tal2a	02g43760	Os.1349.1.S1_at OsAffx.2950.1.S1_s_at	1.25 1.23	1.45 1.32	1.7E-03 5.3E-03	15.87	1.75	21	521	none	-334	-5	nd	+	Ubiquitin carboxyl-terminal hydrolase, family 1, putative, expressed
Tal7	02g43760	Os.1349.1.S1_at OsAffx.2950.1.S1_s_at	1.25 1.23	1.45 1.32	1.7E-03 5.3E-03	16.45	2.03	547	628	341	17	117	+	-	Ubiquitin carboxyl-terminal hydrolase, family 1, putative, expressed
Tal3c	02g47660	Os.7751.1.S1_at	2.25	2.24	1.9E-03	10.93	1.92	53	140	-63	-98	none	-	+	Basic helix-loop-helix, putative, expressed
Tal4c	02g47660	Os.7751.1.S1_at	2.25	2.24	1.9E-03	24.73	3.01	434	367	310	-25	none	+	-	Basic helix-loop-helix, putative, expressed
Tal2c	03g03034	Os.10510.1.S1_at Os.53217.1.S1_x_at	1.49 1.26	3.11 2.83	1.1E-02 6.8E-02	19.55	1.83	0	142	114	-779	6	-	+	Flavonol synthase/flavanone 3-hydroxylase, putative, expressed
Tal3b	03g03034	Os.10510.1.S1_at Os.53217.1.S1_x_at	1.49 1.26	3.11 2.83	1.1E-02 6.8E-02	16.73	2.76	258	759	567	none	none	+	-	Flavonol synthase/flavanone 3-hydroxylase, putative, expressed
Tal11a	03g05370	OsAffx.24978.1.S1_at	10.98	13.02	5.2E-04	17.39	2.74	307	798	331	526	none	+	-	Expressed protein
Tal3c	03g07540	OsAffx.3165.1.S1_at	6.33	3.84	3.6E-02	12.33	2.17	350	248	99	-625	none	-	+	bHLH family protein, putative, expressed
Tal7	03g25490	Os.34992.2.S1_at	2.10	2.02	3.8E-05	16.32	2.01	494	199	30	-363	9	+	-	Cytochrome P450 72A1, putative, expressed
Tal4a	03g37840	Os.20541.1.S1_at	2.24	1.96	2.2E-04	15.58	1.52	0	362	151	-3	none	-	+	Potassium transporter, putative, expressed
Tal2d	04g49194	Os.17316.1.S1_at	22.42	10.49	3.9E-07	8.22	1.26	0	101	26	-715	none	-	+	Naringenin,2-oxoglutarate 3-dioxygenase, putative, expressed
Tal3a	05g12450	OsAffx.26856.1.S1_at	1.81	1.34	2.3E-01	16.07	2.01	294	446	315	none	none	+	-	Hydroquinone glucosyltransferase, putative, expressed
Tal3b	05g27590	Os.57186.1.S1_at	2.40	4.42	3.4E-08	11.40	1.88	2	103	33	-1	none	-	+	Wound-induced protein WI12, putative, expressed
Tal11b	06g14750	OsAffx.15432.1.S1_at	1.29	1.30	2.0E-01	12.44	2.77	129	313	195	160	-15	+	-	Phosphatidylinositol-4-phosphate 5-Kinase family protein, putative, expressed
Tal1c	06g14750	OsAffx.15432.1.S1_at	1.29	1.30	2.0E-01	12.00	2.44	256	178	47	none	none	+	-	Phosphatidylinositol-4-phosphate 5-Kinase family protein, putative, expressed
Tal2a	06g14750	OsAffx.15432.1.S1_at	1.29	1.30	2.0E-01	17.59	1.94	88	79	-22	-618	-33	nd	-	Phosphatidylinositol-4-phosphate 5-Kinase family protein, putative, expressed
Tal4c	06g37080	Os.16282.1.A1_at OsAffx.15788.1.S1_at	5.54 11.84	7.15 9.94	2.7E-10 6.3E-09	14.64	1.78	0	150	39	-1	none	-	+	L-ascorbate oxidase precursor, putative, expressed
Tal8	06g37080	Os.16282.1.A1_at OsAffx.15788.1.S1_at	5.54 11.84	7.15 9.94	2.7E-10 6.3E-09	19.92	2.32	605	661	560	-36	548	+	-	L-ascorbate oxidase precursor, putative, expressed
Tal2g	06g46500	Os.49496.1.S1_at	6.40	6.88	4.3E-08	14.27	2.01	117	89	59	-489	-47	-	+	Monocopper oxidase, putative, expressed
Tal11a	06g47950	OsAffx.15977.1.S1_s_at	1.78	1.67	2.8E-02	16.20	2.55	19	527	none	-328	0	nd	nd	Tetratricopeptide-like helical, putative, expressed
Tal1c	07g06970	Os.49794.1.S1_at	2.95	2.27	1.3E-02	5.97	1.22	0	216	24	none	none	-	+	HEN1, putative, expressed
Tal3a	07g06970	Os.49794.1.S1_at	2.95	2.27	1.3E-02	16.21	2.03	354	930	815	444	none	+	-	HEN1, putative, expressed
Tal4b	07g34510	Os.51294.1.S1_at	0.95	1.00	2.8E-01	8.88	1.63	33	302	151	-425	none	nd	nd	Retrotransposon protein, putative, unclassified, expressed
Tal3b	07g36430	Os.31021.1.S1_at	2.53	2.40	2.6E-02	15.78	2.6	108	117	31	-4	none	-	+	Expressed protein
Tal6	07g47790	Os.8920.1.S1_at	4.16	8.41	3.6E-02	13.38	1.7	8	798	610	-192	694	+	-	AP2 domain containing protein, expressed
Tal4a	09g20220	Os.4759.1.S1_at	2.17	2.38	4.9E-02	28.93	2.83	280	170	139	-751	34	+	-	Glutathione S-transferase, putative, expressed
Tal2d	09g23560	Os.5983.1.S1_at	2.19	5.02	2.8E-01	14.19	2.17	288	525	none	none	93	nd	nd	Dehydrogenase, putative, expressed
Tal6	09g29100	Os.18607.1.S1_at	1.64	1.97	3.6E-02	17.00	2.13	167	0	0	0	0	-	+	Cyclin, putative, expressed
Tal4b	09g32100	Os.16365.1.S1_at	3.34	2.45	8.0E-03	8.15	1.5	16	270	84	21	none	-	+	Expressed protein
Tal1b	10g38489	Os.2612.1.S1_at	4.75	4.14	4.00E-07	8.75	4.13	325	64	20	none	none	nt	nt	glutathione S-transferase GSTU6, putative, expressed
Tal9a	11g01480	Os.18448.1.S1_s_at OsAffx.30765.1.S1_at	5.42 5.74	3.94 4.10	8.2E-06 5.4E-06	19.71	2.56	206	776	621	365	none	+	-	MYB family transcription factor, putative, expressed
Tal9a	12g01490	Os.18448.1.S1_at	5.21	3.92	2.6E-05	19.71	2.56	205	302	191	151	none	+	-	MYB family transcription factor, putative, expressed
Tal6	12g42970	Os.11382.1.S1_at	2.31	1.65	2.2E-04	16.84	2.14	139	132	30	-565	12	-	+	GATA zinc finger domain containing protein, expressed
Tal6	12g42970	Os.11382.1.S1_at	2.31	1.65	2.2E-04	18.27	2.32	411	107	5	-590	-13	-	+	GATA zinc finger domain containing protein, expressed

a Expression values are from the GeneChip expression experiment; see Materials and Methods.

b Prefix “LOC_Os” is omitted.

c Fold change in transcript abundance in leaves at 96h relative to 2h after inoculation with *X. oryzae* pv. oryzicola BLS256 (Xoc).

d Fold change in transcript abundance at 96h in Xoc-inoculated leaves relative to mock-inoculated leaves.

e Calculated for the comparison of transcript abundance in Xoc vs. mock inoculated leaves across all time points.

f Score is according to Doyle et al. [32] except that new RVDs ‘SN’ and ‘YG’, present in Tal2g, were assigned nucleotide association frequencies of ‘NN’ and ‘NG’, respectively (see text)**.**

g EBE relative score, ratio of the observed EBE score to the best possible score for the TAL effector [32].

h EBE rank among the single best scoring sites for the TAL effector in each rice promoter [32].

i Distance in bases from the 5' end of the EBE to the translational start site (TLS) of the target locus; a positive value indicates a location downstream of the EBE.

j Distance in bases from the 5' end of the EBE to the transcriptional start site (TXS) based on cDNA evidence in the Rice Genome Annotation Project Release 7 (http://rice.plantbiology.msu.edu/); a positive value indicates a location downstream of the EBE; none, cDNA evidence of TXS missing.

k Distance in bases from the 5' end of the EBE to the nearest identified putative TATA box; a positive value indicates a location downstream of the EBE; none, putative TATA box not present.

l Distance in bases from the 5' end of the EBE to the nearest identified putative Y patch; a positive value indicates a location downstream of the EBE; none, putative Y patch not present.

m Results of RT-PCR 48h after inoculation, relative to a negative control inoculation (see Supplemental Figure S1); Xoc, *X. oryzae* pv. oryzicola BLS256; Xag, *X. axonopodis* pv glycines EB08; +, induced; -, not induced; nd, transcript not detected by RT-PCR (in each case, amplification by standard PCR from genomic DNA as template was confirmed); nt, not tested.

The corrected Table 1 can be seen here.

The corrected Table S7 can be downloaded here. The legend remains unchanged.

## Supporting Information

Table S7 All computationally predicted targets in rice (cv. Nipponbare) of TAL effectors of *Xanthomonas oryzae* pv. oryzicola BLS256 (Xoc) and TAL effectors of *Xanthomonas oryzae* pv. oryzae PXO99^A^ (Xoo).
(XLSX)
