# Supplementary material for: Code-Assisted Discovery of TAL Effector Targets in Bacterial Leaf Streak of Rice Reveals Contrast with Bacterial Blight and a Novel Susceptibility Gene
Source: PLoS Pathog. 2014 Feb 27;10(2):e1003972. doi: 10.1371/journal.ppat.1003972 (PMC3937315; doi:10.1371/journal.ppat.1003972)
Supplement: Figure S1 — Experimentally verified targets of X. oryzae pv. oryzicola BLS256 (Xoc) TAL effectors in rice: results of RT-PCR analyses to test specific dependence of induction on the TAL effector. Targets (and actin, which was used as an internal control to normalize cDNA amounts) are indicated at far right; “Os_LOC” is omitted from locus IDs. Xanthomonas axonopodis pv. glycines strain EB08 (Xag) was used to deliver individual Xoc TAL effectors and test their sufficiency for induction of respective predicted targets, and Xoc tal gene knock-out mutants were used to test the requirement of each TAL effector for target induction. Xag transformed with, from left to right, vector pAC99 carrying the gene for the TAL effector being tested, another tal gene as a specificity control, or no tal gene (–) were used. For Xoc, from left to right, the wildtype (WT), a marker exchange mutant with a disruption of the gene for the test TAL effector and transformed with the empty vector pAC99, that mutant transformed with pAC99 carrying the intact tal gene (designated in parentheses) cloned in pAC99 for complementation, a type III secretion-deficient Xoc derivative (hrcC−), and an independent tal gene mutant as a specificity control were used, except that no Xoc inoculations were done for Tal2a targets because no tal2a mutant was obtained. Plant tissue for RNA preparation was harvested at 48 h after infiltration and actin was used as internal control to normalize cDNA amounts. Experiments were repeated multiple times including samples collected at 72 h after infiltration and showed identical results. (PDF) [file ppat.1003972.s001.pdf]

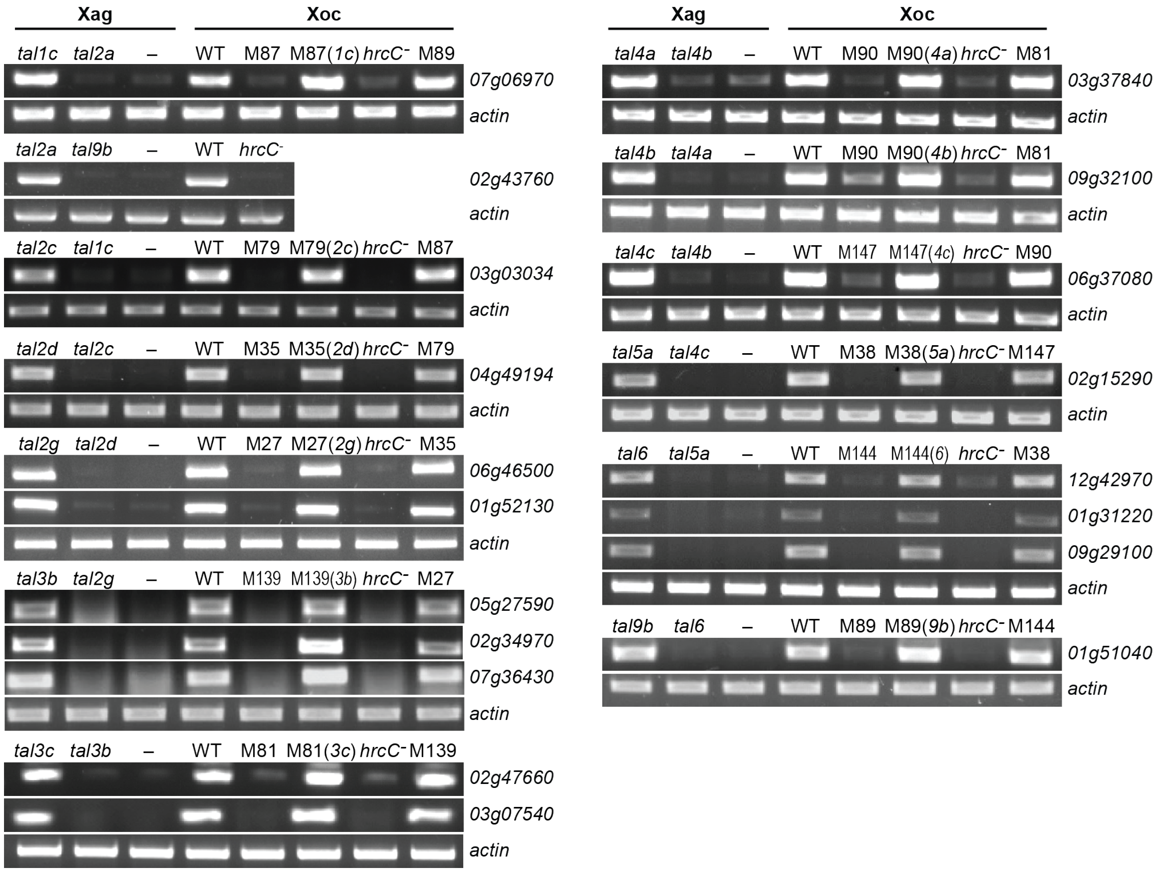

Figure S1. Experimentally verified targets of *X. oryzae* pv. oryzicola BLS256 (Xoc) TAL effectors in rice: results of RT-PCR analyses to test specific dependence of induction on the TAL effector. Targets (and actin, which was used as an internal control to normalize cDNA amounts) are indicated at far right; “Os_LOC” is omitted from locus IDs. *Xanthomonas axonopodis* pv. glycines strain EB08 (Xag) was used to deliver individual Xoc TAL effectors and test their sufficiency for induction of respective predicted targets, and Xoc *tal* gene knock-out mutants were used to test the requirement of each TAL effector for target induction. Xag transformed with, from left to right, vector pAC99 carrying the gene for the TAL effector being tested, another *tal* gene as a specificity control, or no *tal* gene (–) were used. For Xoc, from left to right, the wildtype (WT), a marker exchange mutant with a disruption of the gene for the test TAL effector and transformed with the empty vector pAC99, that mutant transformed with pAC99 carrying the intact *tal* gene (designated in parentheses) cloned in pAC99 for complementation, a type III secretion-deficient Xoc derivative (*hrcC*⁻), and an independent *tal* gene mutant as a specificity control were used, except that no Xoc inoculations were done for Tal2a targets because no *tal2a* mutant was obtained. Plant tissue for RNA preparation was harvested at 48 h after infiltration and actin was used as internal control to normalize cDNA amounts. Experiments were repeated multiple times including samples collected at 72 h after infiltration and showed identical results.
