# Supplementary material for: Code-Assisted Discovery of TAL Effector Targets in Bacterial Leaf Streak of Rice Reveals Contrast with Bacterial Blight and a Novel Susceptibility Gene
Source: PLoS Pathog. 2014 Feb 27;10(2):e1003972. doi: 10.1371/journal.ppat.1003972 (PMC3937315; doi:10.1371/journal.ppat.1003972)
Supplement: Figure S2 — Functionality of Tal2c and Tal2d in the M27 mutant derivative of X. oryzae pv. oryzicola BLS256. Shown is accumulation of transcripts of the Tal2c and Tal2d targets (Table 1 and Table S7), and the two Tal2g targets for reference, in rice at 48 hr after infiltration with wild type (WT), M27, or the type III secretion-deficient hrcC− mutant strain, determined by RT-PCR. Actin transcript was included as a control to normalize cDNA amounts. Experiments were repeated twice showing consistent results. (PDF) [file ppat.1003972.s002.pdf]

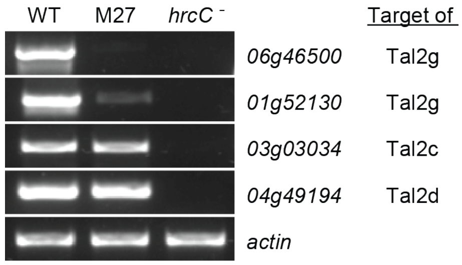

Figure S2. Functionality of Tal2c and Tal2d in the M27 mutant derivative of *X. oryzae* pv. oryzicola BLS256. Shown is accumulation of transcripts of the Tal2c and Tal2d targets (Table 1 and Table S7), and the two Tal2g targets for reference, in rice at 48 hr after infiltration with wild type (WT), M27, or the type III secretion-deficient *hrcC*$^{-}$ mutant strain, determined by RT-PCR. Actin transcript was included as a control to normalize cDNA amounts. Experiments were repeated twice showing consistent results.
