# Supplementary material for: Code-Assisted Discovery of TAL Effector Targets in Bacterial Leaf Streak of Rice Reveals Contrast with Bacterial Blight and a Novel Susceptibility Gene
Source: PLoS Pathog. 2014 Feb 27;10(2):e1003972. doi: 10.1371/journal.ppat.1003972 (PMC3937315; doi:10.1371/journal.ppat.1003972)
Supplement: Figure S3 — Performance of a Naive Bayes classifiers trained on all EBE features or a logistic regression classifier trained on distance to transcriptional start site (TXS) using leave-one-out cross validation. (A) Receiver operating characteristic curve. (B) Precision and recall. (PDF) [file ppat.1003972.s003.pdf]

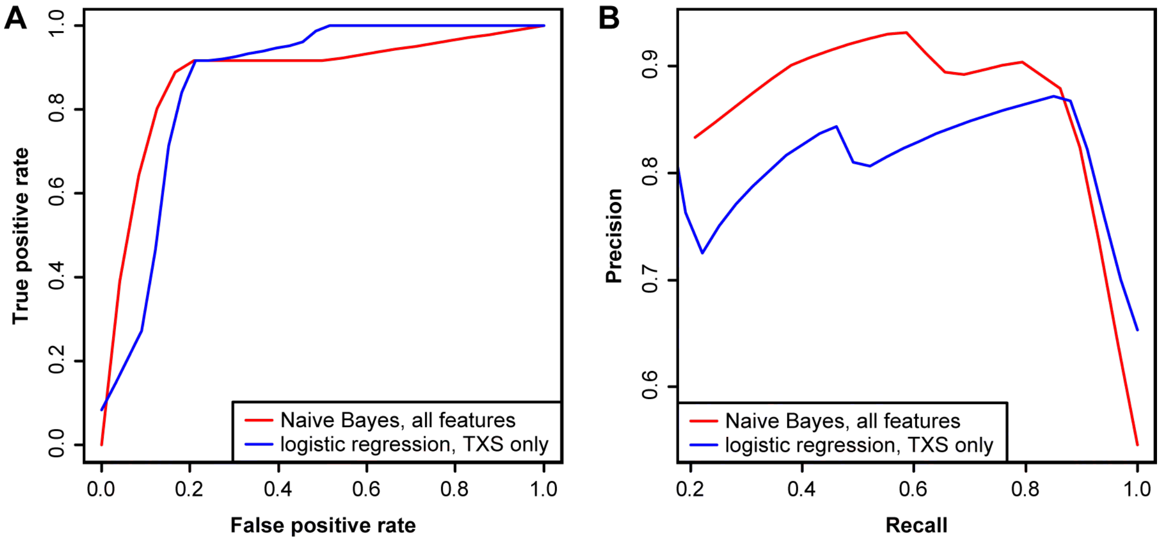

Figure S3. **Performance of a Naive Bayes classifiers trained on all EBE features or a logistic regression classifier trained on distance to transcriptional start site (TXS) using leave-one-out cross validation. (**A) Receiver operating characteristic curve. (B) Precision and recall.
