# Supplementary material for: Code-Assisted Discovery of TAL Effector Targets in Bacterial Leaf Streak of Rice Reveals Contrast with Bacterial Blight and a Novel Susceptibility Gene
Source: PLoS Pathog. 2014 Feb 27;10(2):e1003972. doi: 10.1371/journal.ppat.1003972 (PMC3937315; doi:10.1371/journal.ppat.1003972)
Supplement: Figure S4 — Lower hydrogen peroxide levels in rice leaves infiltrated with X. oryzae pv. oryzicola BLS256 (Xoc) compared to X. oryzae pv oryzae PXO99A (Xoo)- or mock-treated leaves. Hydrogen peroxide activity was determined in 10 cm leaf segments 4 days after infiltration with Xoc, Xoo, or water (Mock), using a chemiluminescence method [1]. The difference between catalase-treated and non-treated samples was considered a relative measure of H2O2. Values are averages of three replicates. Vertical bars show standard deviation. (PDF) [file ppat.1003972.s004.pdf]

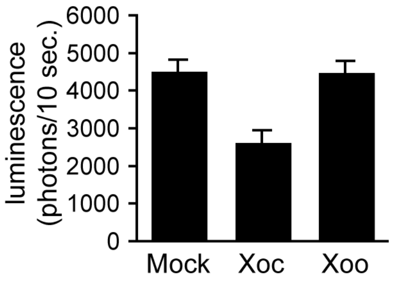

Figure S4. Lower hydrogen peroxide levels in rice leaves infiltrated with *X. oryzae* pv. oryzicola BLS256 (Xoc) compared to *X. oryzae* pv oryzae PXO99^{A} (Xoo)- or mock-treated leaves. Hydrogen peroxide activity was determined in 10 cm leaf segments 4 days after infiltration with Xoc, Xoo, or water (Mock), using a chemiluminescence method [1]. The difference between catalase-treated and non-treated samples was considered a relative measure of H_2O_2. Values are averages of three replicates. Vertical bars show standard deviation.

References

1. Perez F, Rubio S (2006) An improved chemiluminescence method for hydrogen peroxide determination in plant tissues. Plant Growth Regul 48: 89-95.
