# Supplementary material for: Code-Assisted Discovery of TAL Effector Targets in Bacterial Leaf Streak of Rice Reveals Contrast with Bacterial Blight and a Novel Susceptibility Gene
Source: PLoS Pathog. 2014 Feb 27;10(2):e1003972. doi: 10.1371/journal.ppat.1003972 (PMC3937315; doi:10.1371/journal.ppat.1003972)
Supplement: Figure S5 — Effects of the protein synthesis inhibitor cycloheximide (CHX) on expression kinetics of targets of X. oryzae pv. oryzicola BLS256 (Xoc) TAL effectors. Shown are results of RT-PCR performed on rice (cv. Nipponbare) leaf tissue harvested at 0, 8, 16, 24, and 36 hours after infiltration (hai) with Xoc, Xoc plus 50 µM CHX, or 50 µM CHX alone. Targets are indicated at right, by locus ID, omitting the prefix “LOC_Os”. Pathogenesis-related genes PR1a (07g03710), PR1b (01g28450), PAL (02g41630), and EL2 (03g01740), previously observed to be induced by biotic stresses [2]–[4] and 05g42150, the most significantly Xoc-induced gene in our dataset (Table S1) and not predicted to be a TAL effector target, were used as controls for the effect of CHX treatment. An actin gene, which was insensitive to any treatment, is included as a reference for relative transcript abundance across samples. Experiments were repeated once with 50 µM and once with 100 µM CHX using the 24 time point, and showed similar results. (PDF) [file ppat.1003972.s005.pdf]

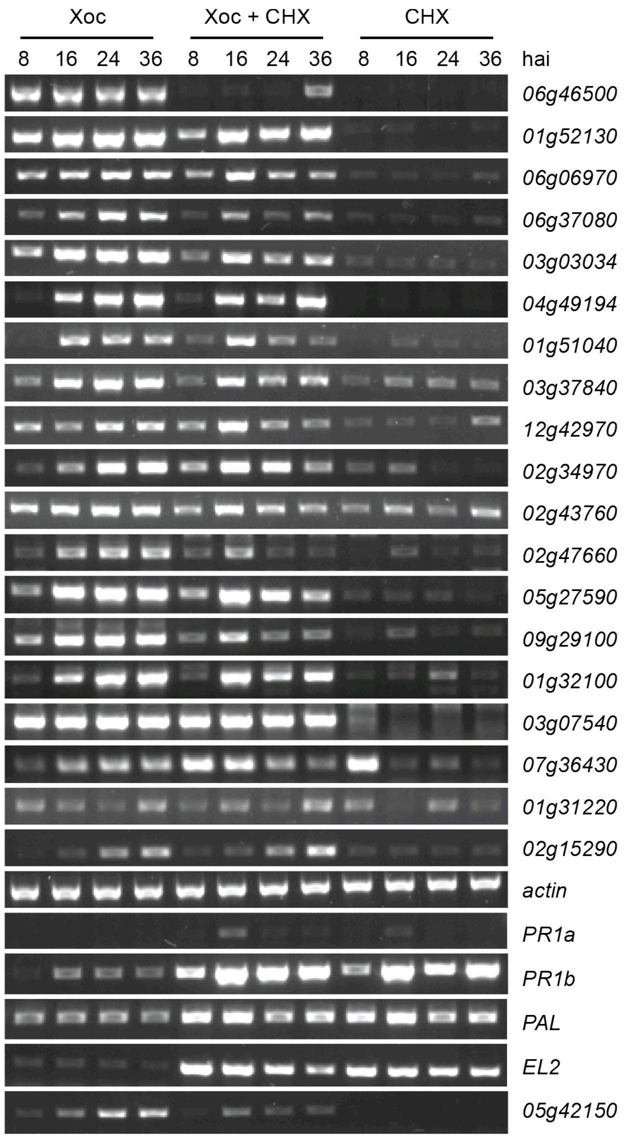

Figure S5. Effects of the protein synthesis inhibitor cycloheximide (CHX) on expression kinetics of targets of *X. oryzae* pv. oryzicola BLS256 (Xoc) TAL effectors. Shown are results of RT-PCR performed on rice (cv. Nipponbare) leaf tissue harvested at 0, 8, 16, 24, and 36 hours after infiltration (hai) with Xoc, Xoc plus 50 μM CHX, or 50 μM CHX alone. Targets are indicated at right, by locus ID, omitting the prefix “LOC_Os”. Pathogenesis-related genes *PR1a* (*07g03710*), *PR1b* (*01g28450*), *PAL* (*02g41630*), and *EL2* (*03g01740*), previously observed to be induced by biotic stresses [1-3] and *05g42150*, the most significantly Xoc-induced gene in our dataset (Table S1) and not predicted to be a TAL effector target, were used as controls for the effect of CHX treatment. An actin gene, which was insensitive to any treatment, is included as a reference for relative transcript abundance across samples. Experiments were repeated once with 50 μM and once with 100 μM CHX using the 24 time point, and showed similar results.

References

1. Wang L, Vinogradov EV, Bogdanove AJ (2013) Requirement of the lipopolysaccharide O-chain biosynthesis gene *wxocB* for type III secretion and virulence of *Xanthomonas oryzae* pv. oryzicola. J Bacteriol.
2. Chen F, Gao MJ, Miao YS, Yuan YX, Wang MY, et al. (2010) Plasma membrane localization and potential endocytosis of constitutively expressed XA21 proteins in transgenic rice. Mol Plant 3: 917-926.
3. Minami E, Kuchitsu K, He DY, Kouchi H, Midoh N, et al. (1996) Two novel genes rapidly and transiently activated in suspension-cultured rice cells by treatment with N-acetylchitoheptaose, a biotic elicitor for phytoalexin production. Plant Cell Physiol 37: 563-567.
