# Supplementary material for: Code-Assisted Discovery of TAL Effector Targets in Bacterial Leaf Streak of Rice Reveals Contrast with Bacterial Blight and a Novel Susceptibility Gene
Source: PLoS Pathog. 2014 Feb 27;10(2):e1003972. doi: 10.1371/journal.ppat.1003972 (PMC3937315; doi:10.1371/journal.ppat.1003972)
Supplement: Figure S6 — Expression patterns of the two targets of Tal2g, Os06g46500 and Os01g52130 , in the GeneChip experiment. Results are plotted as in Figure 2. (PDF) [file ppat.1003972.s006.pdf]

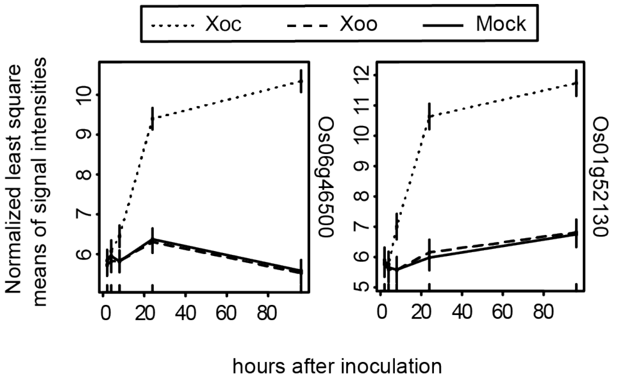

Figure S6. Expression patterns of the two targets of Tal2g, *Os06g46500* and *Os01g52130*, in the GeneChip experiment. Results are plotted as in Figure 2.
