# Supplementary material for: Code-Assisted Discovery of TAL Effector Targets in Bacterial Leaf Streak of Rice Reveals Contrast with Bacterial Blight and a Novel Susceptibility Gene
Source: PLoS Pathog. 2014 Feb 27;10(2):e1003972. doi: 10.1371/journal.ppat.1003972 (PMC3937315; doi:10.1371/journal.ppat.1003972)
Supplement: Text S1 — Supporting information for Materials and Methods. (PDF) [file ppat.1003972.s017.pdf]

Text S1. Supporting information for Materials and Methods.

Nucleotide sequence of the modified 343 bp promoter fragment of *Bs3* (343Bs3) used to test TAL effector activity on different EBEs in the *Agrobacterium*-mediated transient expression-based reporter assay in *N. benthamiana*. PCR primer sites for cloning the 343 bp promoter region of the Bs3 gene (GenBank: EU078684) are highlighted in grey. The substitution of A for C to introduce an *Nco*I site (underlined) is shown in bold. The cognate effector-binding element (EBE) for AvrBs3 is italicized.

>343Bs3 promoter: Modified from *Capsicum annuum* cultivar ECW-30R, GenBank: EU078684

TCATAGTCAAGCTAACGAAACTTATGCAAGGGAAATATGAAATTAGTATGCAAGTAAACTCAAAGAACTA
ATCATTGAACTGAAAGATCAATATATCAAAAAAAAAAAAAAACAATAAAACCGTTTAACCGATAGATTAA
CCATTTCTGGTTCAGTTTATGGGTTAAACCACAATTTGCACACC**A**TGGTTAAACAATGAACACGTTTGCC
TGACCAATTTTATT*ATATAAACCTAACCATCC*TCACAACTTCAAGTTATCATCCCCTTTCTCTTTTCTCC
TCTTGTTCTTGTCACCCGCTAAATCTATCAAAACACAAGTAGTCCTAGTTGCACATATATTTC

Oligonucleotide duplex used to introduce the *Nco*I site into the 343Bs3 promoter by QuickChange mutagenesis (Agilent Technologies).

5′- CCACAATTTGCACACC**A**TGGTTAAACAATGAAC -3′
5′- GTTCATTGTTTAACCA**T**GGTGTGCAAATTGTGG -3′

Oligonucleotide duplexes used to introduce Tal2g EBEs (underlined) into the 343Bs3 promoter at the *Nco*I site.

Tal2g EBE in LOC_Os06g46500
5′-CATGTAGCTGGCAAGTGACCTCAGCTCAGC-3′
3′-ATCGACCGTTCACTGGAGTCGAGTCGGTAC-5′
Tal2g EBE in LOC_Os01g52130
5′-CATGAGAATGGCCCGTAGCCTCTCCTTGTT-3′
3′-TCTTACCGGGCATCGGAGAGGAACAAGTAC-5′
Tal2g EBE in LOC_Os06g13880
5′-CATGGCTTTCGCCAAAAACCAAAGCTCAAG-3′
3′-CGAAAGCGGTTTTTGGTTTCGAGTTCGTAC-5′
Tal2g EBE in LOC_Os12g36920
5′-CATGATAGTAGTTAAAATCCCCAGCTTTGG-3′
3′-TATCATCAATTTTAGGGGTCGAAACCGTAC-5′
Tal2g EBE in LOC_Os05g10650
5′-CATGAGGGTAGCAACTATCGTCAACTAGAT-3′
3′-TCCCATCGTTGATAGCAGTTGATCTAGTAC-5′

RVD sequences of dTALEs used in this study.

dT434: HD NG HD NI NG HD NG HD NI NN NI NG HD HD HD NG NG NG
dT436: HD HD NI NI NI NI HD NN NG NI NI NG NG NG NI NN NI NI NG
dT437: HD NG NN NI NN HD NI HD NI HD NN HD NG NN HD NI HD NN NG NI NI NG
